# Nuclear conversion theory: molecular hydrogen in non-magnetic insulators

**DOI:** 10.1098/rsos.160042

**Published:** 2016-09-07

**Authors:** Ernest Ilisca, Filippo Ghiglieno

**Affiliations:** 1 Matériaux et Phénomènes Quantiques, Université Paris 7 Denis Diderot and CNRS UMR 7162, 75205 Paris Cedex 13, France; 2 Departamento de Fısica, Universidade Federal de Sao Carlos, Caixa Postal 676, CEP 13565-905, Sao Carlos (SP), Brazil

**Keywords:** hydrogen, surfaces, insulators

## Abstract

The hydrogen conversion patterns on non-magnetic solids sensitively depend upon the degree of singlet/triplet mixing in the intermediates of the catalytic reaction. Three main ‘symmetry-breaking’ interactions are brought together. In a typical channel, the electron spin–orbit (SO) couplings introduce some magnetic excitations in the non-magnetic solid ground state. The electron spin is exchanged with a molecular one by the electric molecule–solid electron repulsion, mixing the bonding and antibonding states and affecting the molecule rotation. Finally, the magnetic hyperfine contact transfers the electron spin angular momentum to the nuclei. Two families of channels are considered and a simple criterion based on the SO coupling strength is proposed to select the most efficient one. The denoted ‘electronic’ conversion path involves an emission of excitons that propagate and disintegrate in the bulk. In the other denoted ‘nuclear’, the excited electron states are transients of a loop, and the electron system returns to its fundamental ground state. The described model enlarges previous studies by extending the electron basis to charge-transfer states and ‘continui’ of band states, and focuses on the broadening of the antibonding molecular excited state by the solid conduction band that provides efficient tunnelling paths for the hydrogen conversion. After working out the general conversion algebra, the conversion rates of hydrogen on insulating and semiconductor solids are related to a few molecule–solid parameters (gap width, ionization and affinity potentials) and compared with experimental measures.

## 1. Introduction

Important changes have occurred recently in the experimental measures of the nuclear conversion of hydrogen gases. Hydrogen conversion is now being investigated by a variety of optical and electronic devices [1–3], and the role of hydrogen as a clean and renewable energy carrier has introduced new technological challenges [4].

For a long time, conversion measures were performed from calorimetric or nuclear magnetic resonance methods [5]. Important advances in the study of pure rotational excitation of H_2_ by slow electron impact appeared in 1969 [6] and later on by molecular scatterings on solid surfaces [7]. More accurate methods appeared in 1992 when Raman and infrared spectroscopies could observe the hydrogen conversion by exciting the molecular rovibronic degrees of freedom [8]. As the rotational branches were resolved, it became possible to follow the time evolution of their populations [9,10]. The development of ‘*in situ*’ and ‘site-specific’ methods combines these optical measures with surface spectroscopies, such as X-rays or neutron beams [11], thermal- or photo-induced desorption [12]. They became increasingly efficient in comparing molecules adsorbed on different metal, oxygen or organic surface sites [9,10] or diluted inside semiconductors [13] and insulated cages [14]. In 2003, a Japanese team used a ‘resonance enhanced multi photon ionization (REMPI) method to measure the conversion of hydrogen on a silver surface, combining three techniques: (i) initial preparation of a non-equilibrium hydrogen gas by chromatography and adsorption on Ag at low temperature, (ii) followed by a photo-stimulated desorption, and (iii) a laser time-resolved analysis of the ejected molecules by a two-photon ionization of the molecular electrons [15]. Note that the first two steps involve the interaction of the surface electric fields with the polarizable hydrogen. Recently, new scanning tunnelling microscopy (STM) imaging and optoelectronics devices were able to observe the rotational structure of adsorbed hydrogen, and thus the nuclear spin manifolds [2,3]. Each one of these techniques has brought new and surprising information.

In the case of non-magnetic noble *metals* (and semi-metallic graphite), electron energy low spectroscopy (EELS) observations of fast conversion rates of physisorbed hydrogen allowed the discovery of a new kind of metallic catalysis leaving the molecule free of any bond [16–18]. In the case of *semiconductors*, infrared and Raman devices are also still being used to sample the formation of hydrogen molecules. After annealing, the hydrogen atoms diffuse towards tetrahedral sites where they combine. As soon as the hydrogen molecules form, their nuclear spin populations come into sight and it is possible to follow their slow relaxation towards thermal concentrations over a few hours [19–21]. On a variety of dielectric and diamagnetic *insulators*, different observations by infrared absorption explored the pores of metal--organic frameworks (MOF) filled with hydrogen gas, revealing that oxygen sites could be as effective as the metallic ones to provide fast rates, comparable to the metallic ones of a few minutes [9,10,22]. Ionization of the molecules by REMPI methods corroborated the efficiency of oxygen ions, and attributed the fast *o*–*p* conversion observed on *amorphous* ice to their giant surface electric fields [23].

The hydrogen nuclear spin isomers, with the very long lifetimes, have applications in various contexts for research or industrial purposes. The possible preparation of hydrogen molecules either in a pure state or in non-thermal proportions, together with ‘site-specific’ spectroscopy, brings important information on surfaces [9]. For practical applications, giant nuclear magnetic resonance (NMR) signals, based on the polarization transfer of para molecules, are used in medical imaging [24]. In astrophysics, the ortho–para concentration helps us to date the formation of interstellar clouds [25], and in chemistry, it is used to follow the transient states of chemical reactions [26].

For industry, nuclear conversion appears as an interesting tool to develop nanoporous materials with high selectivity. The main challenge concerns the practical use of the H_2_ internal energy, which requires condensed storage forms [4]. The liquefaction process also uses conversion stages which represent from 18% to 53% of the total liquefaction work owing to the number of stages [27].

To sum up, these numerous observations of hydrogen conversion on a variety of dielectric and diamagnetic solids (metals, insulators and semiconductors) by a variety of techniques (Raman, IR, REMPI and STM) have shown that non-magnetic solids can convert non-equilibrium mixtures of hydrogen, and have a number of applications. Unfortunately, up to now, there has been little established theory to conceptualize the various conversion processes and guide experimentalists.

The link between the nuclear rotational and spin symmetries of molecular hydrogen depends on the electron system in which the nuclei are embedded. In particular, the ortho and para varieties are different nuclear manifolds of the electron ground state. Conversion mixes these nuclear states through an electromagnetic interaction with a solid, and thus breaks the symmetry introduced by the Pauli principle. The old Wigner theory, based on a catalyst magnetic ground state exerting an inhomogeneous magnetic field and torque on the molecule to uncouple the nuclear spin precessions, cannot provide a useful framework to interpret the numerous observations of hydrogen conversion on non-magnetic solids. The difficulties encountered in elaborating a theory large enough to model non-magnetic hydrogen catalysis are conceptual and related to the large variety of observations. Main questions to answer are as follows:

— Is it possible to build a theory that interprets the conversion measures according to the solid properties (insulating or metallic, surface electric gradients, etc.), the scattering geometry and the thermodynamic variables (*T*, *P*, *n*, etc.)?
— How to display and correlate a variety of patterns of different time scales: electronic (from femtoseconds to nanoseconds) to nuclear ones (from about 1 min to hundreds of hours)?
— How to describe and relate in a simple form the very local (over less than 1 Angström) and energetically weak nuclear hydrogen conversion (about 15 meV) to the solid electron fluctuations, which are extended, collective and energetic (over the eV)?

The following theory takes advantage of preliminary ones which require a brief summary. The interpretation of hydrogen conversion on non-magnetic Ag and Cu has opened a new theoretical chapter [28–30]. The work of conversion was shown to emit electron–hole pairs through the solid. The conversion time, measured by REMPI a few years later for Ag(111), confirmed the magnitude of a few minutes predicted by that model [15,25,31]. Infrared and REMPI conversion measures also gave the unique opportunity in 2013–2014 to interpret the relative efficiencies of the metallic and oxygen ions at the surface of MOF structures as well as to compare these oxygen ions with those on amorphous solid water (ASW) [32]. The interpretation considered a real exchange of electrons between the molecule and the solid, able to contact the protons in a two-step process denoted **XY** [33].

The model, herewith described, extends and unifies previous conversion analyses of molecular hydrogen interacting with various non-magnetic solids. It establishes a theoretical ground basis of hydrogen conversion on insulators.

(i) Its main novelty consists in the symmetry requirements analysis. It is concluded that three quantum steps are necessary to break the forbiddenness of the ortho–para transitions when the solid electronic structure is composed of closed shells, well separated from empty open ones by a gap. In terms of interactions, one orbital step is required to change the molecular rotation, a second one connects the non-magnetic ground state to excited ones and the last hyperfine step transfers the electron momenta to the nuclear spins.
(ii) As the magnetic step is not necessarily local (at the molecular site), different dynamical paths are compared. In previous analyses devoted to metallic catalysis (or semi-metals or low gap ionic insulators, or extrinsic semiconductors, etc.), the magnetic excitation was considered extended or collective, i.e. not localized at the molecular site. In such cases, the local conversion operator reduces to a second-order perturbation, but the dynamical cost is high for solids with important gaps, because it is related to high-frequency responses. In the present analysis, these processes are compared to true third-order perturbations characterized by much lower nuclear transition frequencies, no exciton being emitted or adsorbed.
(iii) In order to approach the question of electron delocalization, we extend our previous models in two directions: the first is to increase the electron basis by including the band densities as well as solid and molecular antibonding couplings (and their charge-transfer components). The second extension includes a generalized spin–orbit (SO) coupling inside the electron complex. The influence of SO interaction was first recognized by Sugimoto and Fukutani, who have shown that such a coupling induces a singlet–triplet mixing inside the molecular manifold [23]. Contradistinctly, our general formalism considers the SO interaction inside the full electron complex, but the qualitative and semi-quantitative discussions are more oriented towards SO couplings inside the solid.

The molecule–solid electron basis is described in the first part. It contains a number of one-electron excitations and two-electron exchanges. The basis previously limited to neutral compounds [32,33] is extended to the charge-transfer states, and their mixings are expressed in terms of non-diagonal Coulomb and exchange integrals. In the second part, the spin singlet–triplet magnetic couplings are extracted from the interelectron (SO and exchange) and electron–nuclei (hyperfine contact) interactions. In the third part, the operator algebra expresses the hydrogen nuclear conversion rates induced by non-magnetic catalysts, on the ground basis of time-dependent perturbation theory. Electron and nuclear interactions are mixed, and the fast electronic response is distinguished from the slow nuclear one. In all the considered paths, the irreversible rates are driven by the thermal bath that exchanges *o*–*p* energies released by the nuclear rotation in an attempt of the molecules to accommodate to the surface temperature. The last part is devoted to a discussion of the reaction channels, their nuclear rates and relative efficiencies expressed in terms of a limited number of electronic parameters for different solids.

(Notations, expressions and details of calculations are annexed in the electronic supplementary material).

## 2. ‘Molecule–solid’ electronic structures and interactions

**Figure 1.**
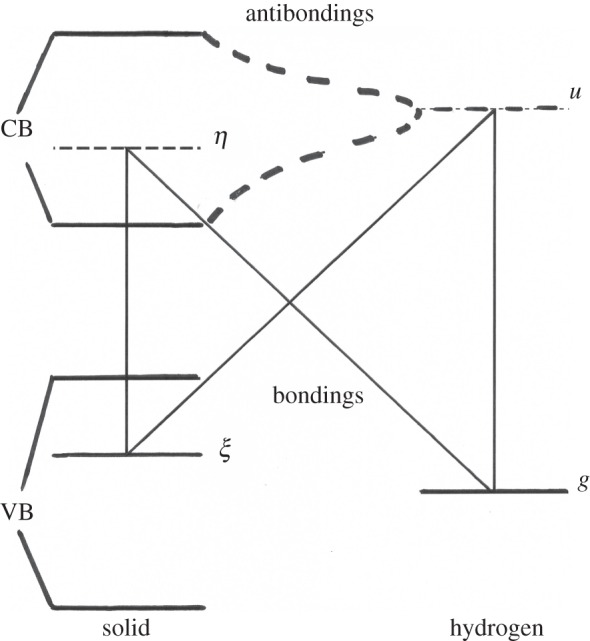
At zero order, the solid electron basis {*α*} is composed of a full valence band (ξ∈VB) and an empty conduction one (η∈CB). The molecular one has a full bonding state: *g* and an empty antibonding one: *u* (*g *= *σ*_g_(1 s) and *u*= *σ*_u_*(1 s)). By solid–molecule perturbations, four bonding–antibonding mixings are operating and involve excitation energies: interbands: $\varepsilon_{\xi \eta }$, purely molecular: $\varepsilon_{gu},$ or charge transfer towards the solid: $\varepsilon_{g\eta }$, or towards the molecule: $\varepsilon_{\xi u}$. (The molecular bonding energy: $\varepsilon_{g}$ lies deeper than represented, $\varepsilon_{g}$ ≈ −15.8 eV). The dashed curve represents the ‘dressing’ of the molecular antibonding *u* state by the conduction electrons.

Our model is simply represented in figure 1, by considering a full valence band and an empty conduction band for a solid at *T *= 0 K. Similarly, the hydrogen molecule orbital basis is limited to a full bonding state and an empty antibonding one. Hydrogen adsorption on surfaces or dilution within the solid breaks the molecular inversion symmetry. In other words, the fundamental hydrogen ground state does not remain completely bonding in the presence of a solid.

It might be possible to account for the local interaction of the molecule with its first solid neighbours, building an interface with the remaining solid acting as a thermal reservoir. It is not our model. We consider an ensemble of identical systems where one molecule interacts with a solid composed of *N* electrons, and thus a total of *N *+ 2 electrons. The interactions between the molecules are thus neglected.

### 2.1. Neutral and ionic antibonding excited states

Molecule and solid fundamental electron eigenstates will be described by pairs of electrons in closed-shell-restricted orbitals. In the hydrogen ground state ${\;}^{1}\Sigma_{g}=|g\bar{g}|$, the bonding *g *= *σ*_*g*_(1 s) molecular orbital is filled by the two electrons in a spin singlet configuration [34]. The required antisymmetry of the two-proton states associates the odd rotational states to nuclear triplet spins, denoted ortho, and even rotational states to singlet spins denoted para. The antibonding state *u* = σ_u_*(1 s), orthogonal to *g* and odd under inversion, associates differently rotational and spin nuclear states of identical parity (for ${\Sigma_{u}}^{+}$ states: J is even (resp. odd) for triplet (resp. even) spins). Most of the bonding density is spherical, whereas the antibonding one is directed along the internuclear axis *ab*. It remains empty for a free molecule. Its progressive filling weakens the hydrogen bond when chemically adsorbed on a surface [35]. It allows important reaction paths in chemisorption, and plays a key role in the conversion of hydrogen interacting with a solid. Such a molecular antibonding state has a strong electron–nucleus hyperfine contact because of its 1 s atomic content of finite amplitude at the protons [36].

The considered solid ground state is composed of a full valence band (VB), where each shell is a doubly occupied Bloch state $\xi =\Psi_{k}(\text{r})$ in the fundamental singlet: ${\;}^{1}\Gamma_{0}=|..\xi \bar{\xi }..|$. The solid excited states denoted *η*, mostly antibonding counterparts of the filled valence bands, build the lowest conduction band CB, empty at *T *= 0. When an electron is promoted from the VB to CB, it leaves a hole behind, while interacting with all remaining electrons [36]. Such excitons might be of singlet or triplet spins owing to the relative orientation of the unpaired electron spins [37]. They differ mostly by an exchange energy. In the following, the considered triplet exciton will be denoted ${\;}^{3}\epsilon (\xi ,\eta )$ (threefold spin degeneracy, *m*_s _= 0, ±1). For example, the parallel orientation (*m*_*s*_ = 1) is represented by the Slater determinant |ξη|.

Molecular and solid electron clouds slightly overlap, and their global complex must be described by eigenstates totally antisymmetric with respect to electron permutation. The fundamental ground state of the molecule–solid electron complex is described at zero order by the antisymmetrized product of the separate ground states: a total singlet, represented by the Slater determinant: ${\;}^{1}\text{G}={\;}^{1}({\;}^{1}\Gamma_{0}\times {\;}^{1}\Sigma_{g})=|..\xi \bar{\xi }..g\bar{g}|$.

In the case of physisorption herewith considered, the molecule–solid repulsions remain much weaker than the antibonding excited energies, and are perturbations of the fundamental ground state. Whereas empty, even at room temperature, the antibonding *u* is strongly admixed with the solid conduction band, because of reciprocal spatial extension and resonant energies [35]. As the first and important sizable and measurable effect, the molecule acquires a small electron dipole and becomes IR active. The internuclear distance is enlarged and fluctuates [3,38]. Some electronic charge might then be transferred to the solid [38] or to the molecule [39].

**Figure 2.**
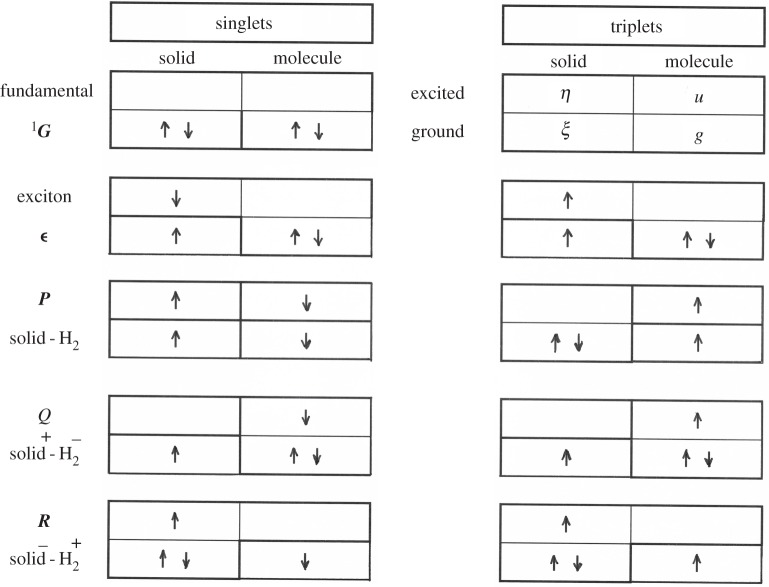
The solid–molecule electron basis is denoted, represented and classified in singlets or triplets, and neutral or charge-transfer states. The first row represents the fundamental ground state orbital occupation and the orbital notation. For each following excited state, whose notation and character are defined in front: the first (resp. second) row represents the bonding (resp. antibonding) occupation states, whereas the first (resp. second) column is relative to the solid (resp. molecular) states.

All one-electron elementary magnetic excitations are herewith considered, either on the solid side or in the molecular manifold. First, consider the tensorial product of the solid and molecule both neutral. Possible excitations towards antibonding states are magnetic (triplets) if they occur inside the solid: ${\;}^{3}\epsilon ={\;}^{3}({\;}^{3}\epsilon \times \Sigma_{g}^{1})$ or inside the molecule: ${\;}^{3}\text{P}={\;}^{3}({\;}^{1}\Gamma_{0}\times {\;}^{3}\Sigma_{u})$; but can also result in a non-magnetic singlet state if both are simultaneous: ${\;}^{1}\text{P}={\;}^{1}({\;}^{3}\epsilon \times {\;}^{3}\Sigma_{u})$. The channel allows an exchange of electrons between the molecule and the solid, already studied [32,33]. However, the complex basis contains also ionic species, obtained by an electron transfer from the solid towards the molecule denoted: ${\;}^{\left[S\right]}\text{Q}={\;}^{\left[S\right]}({\;}^{2}\Gamma_{+}\times {\;}^{2}\Sigma_{u})$ or from the molecule to the solid denoted: ${\;}^{\left[S\right]}\text{R}={\;}^{\left[S\right]}({\;}^{2}\Gamma_{-}\times {\;}^{2}\Sigma_{g}),\;([S]=2S+1)$. ${\;}^{2}\Sigma_{g/u}$ and $\Gamma_{\pm }$ are the fundamental states of respectively $H_{2}^{\pm }$ and the ± ionized solid. Altogether, the herewith considered main electron basis, represented in figure 2, is composed of the fundamental ${\;}^{1}\text{G}$ and the one-electron-excited states of either singlets: ${\;}_{\;}^{1}\text{Q}$, ${\;}^{1}\text{R}$ or triplets ${\;}^{3}\epsilon ,{\;}^{3}\text{P},{\;}^{3}\text{Q},{\;}^{3}\text{R},$ as well as the two-electron-excited singlet ${\;}^{1}\text{P}$. Each one of these excited states contains the number of conduction states. Their wave functions and energies are given in electronic supplementary material, tables A1 and A2.

### 2.2. Electron repulsion and excitation energies

The adsorbed molecule interacts electrostatically with the whole solid, through a surface potential. The neighbour ions can play a particular role mostly when it is an impurity with strong local fields. However, all electrons are indistinguishable, and band states are delocalized through the solid. The three families of excited compounds Γ (neutral *P*, positively *Q* or negatively *R* ionized molecule) are differently incorporated by the molecule–solid electron repulsion, inside the fundamental configuration. Each admixture is expressed by a ratio between the repulsion strength and the corresponding excitation energy: $\left|{\;}^{1}G\rangle ={\left|{\;}^{1}G\right\rangle }_{0}+\sum_{\Gamma }\{C(G\Gamma )/(E({\;}^{1}\Gamma )-E({\;}^{1}G))\}|{\;}^{1}\Gamma \right\rangle$. The Coulomb repulsion denoted **C**, among the solid and molecular electrons, can be enlarged to a sum over all electrons: $\text{C}=\sum_{\alpha \neq \beta }(1\text{/}r_{\alpha \beta })$, because internal repulsions are vanishing in the non-diagonal elements. As it conserves the total electronic spin, it is natural to distinguish between two channels denoted singlet or triplet. Within the considered basis, represented in figure 2, the non-diagonal Coulomb interaction expands as

$$\text{C}=\sum_{\Gamma }C(\epsilon \Gamma )\{|{\;}^{3}\epsilon \rangle \langle {\;}^{3}\Gamma |+|{\;}^{3}\Gamma \rangle \langle {\;}^{3}\epsilon |\}+C(G\Gamma )\{|{\;}^{1}G\rangle \langle {\;}^{1}\Gamma |+|{\;}^{1}\Gamma \rangle \langle {\;}^{1}G|\},$$

and thus links the excited Γ-states either to the ground ${\;}^{1}G$ or to the exciton ${\;}^{3}\epsilon$ states. The coefficients C(GΓ) and C(ϵΓ), expressed in electronic supplementary material, table A3, depend upon the relative positions of the molecular electrons and protons with respect to the solid, and should be estimated in function of the molecule–solid distance. Relative admixtures depend also upon the position of the solid gap with respect to the molecular bonding–antibonding (*b*–*ab*) one: $\varepsilon_{gu}$, as discussed in the last section. The solid eigenstates are naturally expressed in the laboratory frame, whereas the molecular ones *g* and *u*, are referred to a frame attached to the molecule.

First, consider an electron exchange between the molecule and the solid where an electron occupying the bonding orbital *g* jumps onto an empty solid orbital *η*, whereas an electron occupying a solid orbital *ξ* jumps on the antibonding molecular orbital *u* (figure 1). The Coulomb orbital matrix elements induce the exchange: $\left\langle \xi \eta |1\text{/}r_{12}|gu\right\rangle$ depends upon the molecular orientation, because the antibonding orbital is linked to the internuclear axis

$$\text{u}(\text{r})=u(r){\text{Y}}^{1}(\hat{r}).\;{\text{Y}}^{1}(\hat{ab})={\text{u}}^{1}(\text{r}).\;{\text{Y}}^{1}(\hat{ab}).$$

Each component of the antibonding tensor $u^{1}(\text{r})$ connects the ortho and para varieties with different rotational states: $\left\langle j_{p}m_{p}|u(\text{r})|\;j_{o}m_{o}\rangle =a_{op}u_{m_{op}}^{1}(r\right)$; the rotational constant $a_{op}$ being expressed in electronic supplementary material, A2.1 and $m_{op}=-m_{p}+m_{o}$ [40]. With respect to the solid, and considering the *o*–*p* link, the molecular antibonding eigenstate *u* behaves as if it had three components of *p* form: *u_x_*, *u_y_*_,_ *u_z_* oriented along three orthogonal axes attached to the solid structure. Thus, the Coulomb spatial repulsion between two molecular and two solid orbitals, each made of a product of bonding and antibonding electrons, connects rotational states of different parities. Moreover, although it conserves the total electron spin, it frequently changes the individual solid and molecular ones. For instance, the excited singlet ${\;}^{1}({\;}^{3}\epsilon \times {\;}^{3}\Sigma_{u})$ connected to the fundamental ${\;}^{1}G$ is a product of triplets, whereas the triplet ${\;}^{3}({\;}^{1}\Gamma_{0}\times {\;}^{3}\Sigma_{u})$ connected to the ${\;}^{3}\epsilon$ exciton is a product of a singlet and a triplet.

Let us now consider the ionic excited states (the *Q* and *R* states defined in figure 2 and electronic supplementary material, tables A1–A2) which are all products of doublets (*s *= 1/2). The Coulomb integrals involve now three eigenstates of one medium and one of the other, either solid or molecular. The *Q* states use the antibonding *u* state through the repulsions $\left\langle \xi \eta |1\text{/}r_{12}|\xi u\rangle \text{ and }\langle g\eta |1\text{/}r_{12}|gu\right\rangle$ where one electron is in a mixed product of antibonding states *uη* and the other one in a covalent bond, either in the localized molecular ground or in the valence band. Both link different rotational states and establish an important and necessary step in the nuclear transition. Differently, the *R* states use the spread of the solid antibonding states *η*, but the repulsion with the molecular ground *g* state: $\left\langle gg|1\text{/}r_{12}|g\eta \right\rangle$ is slightly weaker. Owing to the spherical shape of *g*, that repulsion is insensitive to the rotational molecular state.

## 3. Electronic and nuclear magnetic interactions

In this section, the interelectron (SO and exchange) and electron–nuclei (hyperfine contact) interactions are summarized [41,42], and their spin singlet–triplet magnetic couplings are extracted.

### 3.1. Singlet–triplet spin–orbit couplings

It is not possible to assign a particular spin to a surface site, because the solid electrons are completely delocalized. At zero order and low temperature, the whole system is considered to be in a singlet state: *S *= 0. In this section, we consider very small perturbations that break such a non-magnetic symmetry, and introduce triplet states: *S *= 1. The described formalism uses restricted Hartree–Fock spin–orbitals, but core polarization manifests through the interaction of an outer electron (e.g. excited to the conduction band) with the remaining electrons in the shell and thus of the same symmetry. Such differences in the otherwise identical radial function of the closed shell are induced by exchange terms and included as they appear in energy expressions for HF wave functions modulated by their orbital densities. Detailed calculations would better incorporate unrestricted UHF wave functions which are flexible enough to accommodate the fact that the radial wave functions of two electrons differing in spin are different [34]. Note that most orbital operators are vanishing in a closed shell, but have non-zero matrix elements between an electron in an open shell and the electrons in the closed shell (or with a hole left behind). In the following, a general one-electron singlet–triplet operator form is used to represent the spin polarization and SO couplings

$$\Lambda =\sum_{\alpha }\Lambda (\alpha ).\text{s}(\alpha ),$$

assuming that an important part of the two-electron couplings have been included inside through a mean field average. Singlets and triplet states are then connected by the following Hamiltonian

$$\Lambda =\sum_{\Gamma }\Lambda (\Gamma \Gamma )\{|{\;}^{1}\Gamma \rangle \langle {\;}^{3}\Gamma |+|{\;}^{3}\Gamma \rangle \langle {\;}^{1}\Gamma |\}+\Lambda (\text{G}\Gamma )\{|{\;}^{1}G\rangle \langle {\;}^{3}\Gamma |+|{\;}^{3}\Gamma \rangle \langle {\;}^{1}G|\}.$$

Spin and spatial parts separate out, and all *N *+ 2 electron averages are expressed in electronic supplementary material, table A4 as products of one-electron orbital matrix elements with the spin difference operator σ (between two spin momenta, as defined in electronic supplementary material, A1.2). The excited triplets of the basis ^3^Γ(Γ* *= *P*, *Q*, *R*, ϵ as expressed in figure 2) are connected to the singlets: either to the excited ones ${\;}^{1}\Gamma :\Lambda (\Gamma \Gamma )=\langle {\;}^{3}\Gamma |\Lambda |{\;}^{1}\Gamma \rangle =\Lambda (\gamma \gamma^{\prime }).\;\sigma$, or to the fundamental one: ${\;}^{1}G:\;\Lambda (G\Gamma )=\langle {\;}^{1}G|\Lambda |{\;}^{3}\Gamma \rangle =\Lambda (\theta \gamma ).\;\sigma$. In the main text, the SO interactions that mix valence and conduction states of the solid (ξη) are assumed to be the leading ones. The interaction couples the orbit of the excited electron to the unpaired electron left behind, or equivalently to the hole. The resulting admixture will be denoted by small letters: $\lambda (\xi \eta )=\Lambda (G\epsilon \text{)/}\varepsilon_{\xi \eta }=(-1\text{/}\sqrt{2})\langle \xi |\Lambda |\eta \rangle \text{/}\varepsilon_{\xi \eta }$. Those within the conduction band (ηη) and the molecular space will be taken into account in electronic supplementary material, A3.2. The one-electron angular momenta constructed from these *S*−*T* mixings are rather sensitive to electron overlaps, configuration interactions and internal electric fields. The two-body SO interactions are more sensitive to the internuclear distances in the solid and thus also couple the electron system to the thermal bath. At low temperature, they maintain the solid in its singlet ground state.

### 3.2. Electron–nucleus hyperfine contacts

The hyperfine contact between the electrons *α* and the hydrogen protons *p* allows a transfer of angular momentum between the nuclei and the electrons [41]. Its antisymmetric part with respect to inversion introduces the nuclear spin difference: ${\text{i}}^{1}={\text{i}}^{1}(a)-{\text{i}}^{1}(b)$, which is the key nuclear spin operator that induces the molecular *o*–*p* conversion

$$\text{Y}=2\zeta \sum_{\alpha ,p}\delta (r_{\alpha p}){\text{s}}^{1}(\alpha )\;.\;\;{\text{i}}^{1}(p)=\zeta {\text{i}}^{1}.\sum_{\alpha }\{\delta (r_{\alpha a})-\delta (r_{\alpha b})\}{\text{s}}^{1}(\alpha )+\cdots$$

${\text{r}}_{\alpha p}$ is the distance vector between proton *p* = *a* or *b*, and electron *α*. The nuclear spin momentum **I** is no longer a good quantum number, but the hyperfine admixture is very weak: $\zeta =3.4\times 10^{-7}$ arb. units. **Y** can induce a simultaneous singlet–triplet transition in the electron and nuclear spin manifolds, and within the considered basis connects the singlet excited states ${\;}^{1}\Gamma$ to the triplet exciton ${\;}^{3}\epsilon$, and the triplet ones ${\;}^{3}\Gamma$ to the fundamental singlet ${\;}^{1}G$.

$$\text{Y}=\sum_{\Gamma }\text{Y}(\Gamma \epsilon )\{|{\;}^{1}\Gamma \rangle \langle {\;}^{3}\epsilon |+|{\;}^{3}\epsilon \rangle \langle {\;}^{1}\Gamma |\}+\text{Y}(G\Gamma )\{|{\;}^{1}G\rangle \langle {\;}^{3}\Gamma |+|{\;}^{3}\Gamma \rangle \langle {\;}^{1}G|\}.$$

Altogether, the *S*/*T* contact Hamiltonian can be written as a product between spin operators and orbital averages Y(GΓ)=Y(θγ)i. σ and Y(ϵΓ)=Y(ϵγ)i. σ. The electron and nuclear spin difference operators **σ** and **i** induce the singlet–triplet and *o*/*p* transitions, as already introduced and defined in electronic supplementary material, A1.2 and A2.2. The matrix elements are expressed in electronic supplementary material, table A5 as functions of the electron densities at the proton positions. For the neutral excitations *P*, *Y*(GP) and Y(ϵP) are proportional to *Y*(*gu*) = *ζ g*(*a*)*u*(*a*). It is the strongest contact interaction, where the hydrogen electrons reach the protons because of their 1 s character. *g*(*a*) [resp. *u*(*a*)] is the amplitude of the *g* [resp. *u*] wave function at the proton a. For the ionic states *Q* and *R* (represented in figure 2), different hyperfine contacts appear between the molecular protons and the solid electrons. When one solid electron jumps back and forth on the molecular antibonding *u*, building the ionized state ^2^Σ_*u*_ of ${\text{H}}_{2}^{-}$ through the excitation *Q*, it reaches the protons efficiently. The contact interactions *Y*(*GQ*) and Y(ϵQ) are then proportional to *Y*(*ξu*) = ζ *ξ* (*m*)*u*(*a*) and *Y*(*ηu*) = *ζη*(*m*)*u*(*a*), and thus to the amplitudes of either the valence state *ξ* or the conduction one *η*, at the molecular centre *m*. Such a contact interaction is increasing exponentially with decreasing ‘molecule–solid’ distance. When one molecular electron jumps back and forth on the solid, building the ionized state ^2^Σ_*g*_ of ${\text{H}}_{2}^{+}$ through the excitation *R*, it reaches also the protons, but differently. The contact interactions *Y*(*GR*) and Y(ϵR) are, in such transfers to the solid, functions of the solid eigenstates' inhomogeneity in the molecular region: Y(gγ)≅ζ.g(a)ab.∇γ(m) for γ=ξ,η. (Such contacts are proportional to the gradient amplitudes of either the valence *ξ* or conduction *η* state at the molecule centre *m*.)

It is interesting to underline that all even–odd (*o*–*p*) couplings, induced by spin operators, are simultaneously singlet–triplet mixing and antisymmetric with respect to inversion.

## 4. Electronuclear conversion rates

### 4.1. Reaction paths

The *o*–*p* conversion of molecular hydrogen involves both a symmetry breaking and a thermal accommodation with the solid. Any *o*–*p* transition necessitates the change of both the rotational and spin nuclear momenta Δ*J* = Δ*I *= 1. The electron system provides paths through transient magnetic states that break the Pauli electron and nuclear antisymmetries and correlate the electron fluctuations to the molecule and phonon dynamics. In most cases, the molecular angular momenta are exchanged with the solid. However, the rotational energies can be shared between the phonons and the molecular motion during the (inelastic) scattering.

When the solid is magnetic, the nuclear changes might occur in one single and real step [30]. For the case of non-magnetic solids herewith considered, two or three virtual steps are necessary. The conversion rate is much slower when induced by non-magnetic solids than for magnetic catalysis; from about a few minutes on noble metals and ionic insulators, such as MOFs [8] or ASW [15], to a few hours in silicon and insulating nanocages [13]. These rates are also rather sensitive to the temperature. There is, thus, a need for a theory large enough to explain the variety of observations and orders of magnitude. When the molecular motion is being trapped inside small nanocages of hard walls, the centre of mass position and momentum become quantified and correlate to the internuclear vibration and rotation as well as with the solid phonons.

The considered ensemble system is composed of four quantum systems interacting with a thermal bath, either continuously or repeatedly owing to the scattering configuration. Two of these systems are molecular, and two are solid ones, and for each: one is orbital, whereas the other one occurs in spin space. The rotational transitions are performed by the electron orbital distribution, whereas the nuclear spin transitions are induced by the spin--orbital electron density. The unperturbed Hamiltonian **H_0_** of the separated molecule and solid also includes an average potential in which the molecules are embedded during the scatterings (in particular all one-centre Coulomb attractions and repulsions as well as an average electron repulsion). The motion of the molecular centre of mass in the solid potential, often erratic, will be described here, by a random classical variable function of time. The **H_0_** quantum eigenbasis, expressed in electronic supplementary material, table A1 and represented in figure 2, is constructed from Slater determinants of the spin--orbital eigenstates. The total interacting system is described by a Hamiltonian that includes corrections that are weak with respect to the energies of the molecule and solid excited states and thus treated as perturbations. At most of the experimental temperatures, it is assumed that the valence band is full, and the conduction band is empty. The perturbation contains two parts: one is the solid fine structure Λ, the other is the solid–molecule interaction **V**

$$\text{H}-{\text{H}}_{\text{0}}=\Lambda +\text{V}.$$

The fine structure Hamiltonian Λ is a function of the orbital position ${\text{r}}_{\alpha }$, momentum ${\text{p}}_{\alpha }$ and spin ${\text{s}}_{\alpha }$ of the electron complex {*α*}. In the context of this study, it includes any electron spin singlet–triplet operator that breaks the non-magnetic character of the solid–molecule ground state. The electromagnetic interaction **V** is composed of two parts: the Coulomb interaction **C** between the solid and the molecular electrons, and the magnetic hyperfine contact **Y** between all the electrons and the hydrogen nuclei.

V=C+Y.

**V** is the function of the position and spin: *r_α_*, *s_α_* of all the electrons *α*, and of the protons *p* = *a*, *b*: internuclear orientation **ab** and spins **I**(*a*) and **I**(*b*). In the following, the *o*–*p* solid–molecule electromagnetic interaction results from the simultaneous action of **C** and **Y**. The position and momenta variables of the solid nuclei, as well as of the molecular centre of mass position: *d*, span the thermostat system. The irreversible flow towards equilibrium is conveniently described by the master equation for populations that governs the time evolution of the hydrogen populations (electronic supplementary material, A2.1), whose rotational energies are: $\varepsilon_{J}=BJ(J+1)$, if their splitting is neglected.

Whatever the path ℘ considered in the following, the *o*–*p* transition probabilities between the rotational manifolds $j_{p}$ and $j_{o}$ are expressed as a product between a quantum probability and a spectral density: J(ε).

$$W_{℘}(j_{o},j_{p})=|{\text{O}}_{℘}(j_{p};j_{o})|{\;}^{2}\;J(\varepsilon_{℘}).$$

It will be assumed that the numerous interactions combine together, and that each path ℘ has the same time dependence function of the hydrogen centre of mass random position. **O** is an electromagnetic operator that connects the hydrogen protons to the electron distribution, and is estimated at an effective solid–molecule distance *d*, which is not necessarily an equilibrium position. In most cases, the molecules collide repeatedly against the solid wall, enter and leave the zone of strong repulsions where the interactions are correlated [39]. Moreover, the ensemble average of time correlation functions is assumed stationary for simplicity, and the different paths are autocorrelated

$$\langle O_{℘}(t^{\prime })O_{{℘}^{\prime }}(t+t^{\prime })\rangle =|{\text{O}}_{℘}(d)|{\;}^{2}\delta_{℘,{℘}^{\prime }}f(t).$$

The corresponding spectral density $J(\varepsilon_{℘})=\int_{0}^{\infty }\text{d}t\;f(t)\;{\text{e}}^{-\text{i }\omega_{℘}t}$ is the Fourier transform of the correlation function *f*(*t*) at the excitation frequency of the path $℘:\varepsilon_{℘}=\hbar \omega_{℘}$.

In the following, we present, discuss and compare two families of processes able to convert the hydrogen molecules when colliding with a non-magnetic insulating solid. In the first denoted **CY**, **C**oulomb-h**Y**perfine contact, the collision transfers the nuclear rotational and spin angular momenta in two steps by emitting magnetic excitons. These are emitted at the local sites, but diffuse through the solid where they relax quickly. In that case, the nuclear *o*–*p* rate is obtained from the high-frequency response of the electron system, more precisely at energies equal to or larger than the electron gap (1–10 eV), and therefore denoted electronic conversion. The second type of **S**pin–**O**rbit-**C**oulomb-h**Y**perfine contact **SOCY** processes transfer the hydrogen nuclear momenta to the solid electron fine structure in three steps. The coupled action of the Coulomb--contact **CY** and the **SO** interactions introduces a small magnetic component in the electronic ground state, allowing the nuclear spin flow. The corresponding conversion rate is then represented by a low-frequency response of the electron system to the nuclear imbalance, at the rotational energies: 15–50 meV, and therefore denoted nuclear conversion.

### 4.2. Electronic conversion

First, consider the high-frequency ortho–para response, denoted Coulomb–contact process: **CY** resulting from the simultaneous action of **C** and **Y**. The Coulomb interaction manifests its effect through either an exchange **C = X**, or a transfer towards the molecule **C = U**, or towards the solid **C = V**, connecting the excited states *P*, *Q* and *R* to the ground and ‘excitonic’ states (see figures 1 and 2 and the non-diagonal elements of electronic supplementary material, table A3). Each transfer is complemented by a different contact **Y** between the electrons and the nuclei (electronic supplementary material, table A5). During the scattering, the molecule–solid potential is known to fluctuate. For example, numerical simulations for hydrogen in silicon indicate energy transfers of the order or larger than 0.5 eV during every few hundredths of femtoseconds [38]. On amorphous structures or unsaturated ionic compounds, the electron fields are found rather high and inhomogeneous, stronger than 10^10 ^Vm^−1^ at a few atomic units of physisorption equilibrium distances [1,23], and even higher each time the molecule collides the hard wall repulsive part of the potential. For each electron transfer to the conduction band: ξ→η, an exciton is emitted and diffuses through the solid. Whatever the molecule–solid Coulomb repulsion of **X, U** or **V** types being the most effective, the hydrogen conversion is sensitive to the triplet manifold of that exciton denoted ${\;}^{3}\epsilon (\xi \eta )$, and each of these electric channels is associated with a particular magnetic hyperfine contact interaction **Y**. Altogether, such processes induce real singlet–triplet spin transitions, both inside the nuclear (Δ*I *= 1) and electron (Δ*S *= 1) manifolds. The resulting conversion rates sum all possible creation of excitons with energies $\varepsilon_{\xi \eta }$:

$$W(j_{o},j_{p})=\sum_{\xi \eta }|{\text{T}}_{\xi \eta }(j_{p};j_{o})|{\;}^{2}J(\varepsilon_{\xi \eta }).$$

The strength measures the simultaneous *S*/*T* and *o*/*p* transitions induced by an electronuclear Hamiltonian **T**

$$|{\text{T}}_{\xi \eta }(j_{p};j_{o})|{\;}^{2}=\sum_{m_{s},m_{p},m_{o},i_{o}}|\langle G;p|\text{T}|{\;}^{3}\epsilon (\xi \eta );o\rangle |{\;}^{2}.$$

To express such an operator **T** through second-order perturbation theory, it is useful to introduce first the Green operator ${\text{G}}_{s}$ that sums the projectors over all the antibonding excited states, but here again limited to Γ = *P*, *Q*, *R* of spin manifold *S* (0 or 1) and energy $\varepsilon_{\Gamma }$ represented in figure 2, because of their (1 s) content and thus of appreciable contact with the nuclei

$${\text{G}}_{s}=\sum_{\Gamma ,m_{s}}\frac{\left|\Gamma ,Sm_{s}\rangle \langle \Gamma ,Sm_{s}\right|}{\varepsilon_{\Gamma }}.$$

**T** combines the action of **C** and **Y** and induces the transfer of angular momenta through the singlet and triplet electron channels

$$\text{T}(o↔p,S↔T)=\sum_{S=0,1}({\text{C G}}_{s}\text{Y}+\text{Y}\;{\text{G}}_{s}\text{C}).$$

The Coulomb step **C** changes both the molecule and solid electron spins, but not the total one. The hyperfine contact **Y** changes the molecular electron and nuclear spins. Together they induce a double ‘flip-flop’ of the solid electron and molecule nuclear spins: the **T** matrix elements between the fundamental ground state and the triplet excitons ${\;}^{3}\epsilon (\xi \eta )$ are calculated and expressed as a product of two scalar products [40], one operating in real space and the other in the spin one

$$\langle {\;}^{1}G|\text{T}|{\;}^{3}\epsilon \rangle =\langle {\;}^{1}G|\text{C}\;{\text{G}}_{0}\text{Y}+\text{Y}\;{\text{G}}_{1}\text{C}|{\;}^{3}\epsilon \rangle =[{\left\{\text{Cy}\right\}}^{1}(\xi \eta ).{\text{Y}}^{1}(\hat{ab})][{\text{i}}^{1}.\;\sigma^{1}].$$

The first term couples the molecular rotation to the electron density, whereas the second one couples the electron and nuclear spins, inducing the double singlet–triplet spin transition (${\text{i}}^{1}$ and $\sigma^{1}$ are the nuclear and electron singlet–triplet tensors defined in electronic supplementary material, A1.2 and A2.2). The summation over the antibonding excited states Γ = *P*, *Q*, *R* is included inside the first-order orbital tensor: ${\left\{\text{C}.\text{y}\right\}}^{1}$, collecting the interactions arising from electron exchanges: *X*, and transfers: solid **→** molecule: *U* and molecule **→** solid: *V*. The repulsion–contact tensor ${\left\{\text{C}.\text{y}\right\}}^{1}$ is precisely defined as

$$\begin{array}{rl} {\left\{\text{C}.\text{y}\right\}}^{1}(\xi \eta ) & =\left\{\left[\frac{{\text{x}}^{1}(\xi \eta )}{\varepsilon_{gu}}+\frac{{\text{x}}^{1}(\eta \xi )}{\varepsilon_{gu}+\varepsilon_{\xi \eta }}\right]Y(gu)-\frac{{\text{U}}^{1}(\xi u)Y(\eta u)+{\text{U}}^{1}(\eta u)Y(\xi u)}{\varepsilon_{\xi u}}\right.\\ & \;+\left.\frac{V(g\xi ){\text{Y}}^{1}(g\eta )+V(g\eta ){\text{Y}}^{1}(g\xi )}{\varepsilon_{g\eta }}\right\}. \end{array}$$

Each electrostatic Coulomb integral (*X*, *U* or *V*) is indexed by the interacting orbitals and weighted by the complementary magnetic contact amplitudes (*Y*/ε), as tabulated in electronic supplementary material, table A5. For low gap semiconductors: $\varepsilon_{gu}\gg \varepsilon_{\xi \eta }$, the first exchange term reduces to ${\text{x}}^{1}(\xi \eta )Y(gu\text{)/}\varepsilon_{gu}$, with ${\text{X}}^{1}(\xi \eta )=({\text{x}}^{1}(\xi \eta )+{\text{x}}^{1}(\eta \xi ))\sqrt{2}$. The excitation energies are either purely molecular: $\varepsilon_{gu}$ or crossed between the solid and the molecule: $\varepsilon_{\xi u}$ or $\varepsilon_{g\eta }$, as represented in figure 1 and defined in electronic supplementary material, table A2. The exponent index 1, at the right of the various tensors, denotes the three components of first-rank spherical tensors: ${\text{Y}}^{1}(\hat{ab}),{\text{X}}^{1},{\text{U}}^{1}\ldots$ which have three components corresponding to the three orientations of the molecular antibonding orbitals ${\text{u}}^{1}(\text{r})=u(r){\text{Y}}^{1}(\hat{r})$. However, for the molecule **→ **solid scalar repulsion *V*, the hyperfine contact induces the rotational transitions, with elements proportional to the electron wave gradients $\nabla^{1}\xi$ or $\nabla^{1}\eta$. The *o*–*p* nuclear matrix elements of such an electron ‘Coulomb–contact’ transition operator, relative to the emission of the exciton ${\;}^{3}\epsilon (\xi \eta ),$ are obtained as

$$\langle \text{p}|\langle {\;}^{1}G|\text{T}|{\;}^{3}\epsilon \rangle |\text{o}\rangle =a_{op}\;\sigma_{i_{o}}^{1}{\left\{\text{C}.\text{y}\right\}}_{m_{op}}^{1}(\xi \eta ).$$

Note that the *electron* tensors have components indexed by *nuclear* quantum numbers: $m_{\mathrm{op}}=m_{o}-m_{p}$ for the orbital ones and *i*_o_ for the spins. They display the connection between the molecular rotation and the electron orbital motion as well as the one between the electron and nuclear spins. (The rotational matrix element $a_{op}$ is defined in electronic supplementary material, A2.1.) Let us now assume that the molecule antibonding orbital *u* is directed towards the solid, and choose the axis *z* to express the transition rates relative to the considered CY(ξη) channels between a couple of ortho and para states

$$W_{e}(j_{o},j_{p})=a_{op}^{2}\sum_{\xi \eta }|C_{e}(\xi \eta )|{\;}^{2}J(\varepsilon_{\xi \eta }).$$

$C_{e}=\sqrt{3}{\left\{\text{C}.\text{y}\right\}}_{0}^{1}\text{/}\hbar$, precisely defined by the *z* component of the orbital tensor ${\left\{\text{C}.\text{y}\right\}}^{1}(\xi \eta ),$ represents an effective Coulomb–contact interaction. Divided by ℏ, it has the dimension of a frequency. Such matrix elements, as well as subsequent transition rates, generalize the exchange-contact **XY** process presented in previous papers [32,33], and by extending the basis to one-electron molecule–solid charge-transfer states (*Q* and *R*) add the **UY** and **VY** conversion channels (*C* = *U* or *V*). The solid–molecule electron exchange **XY** channel (*C *= *X*) has been studied to interpret the infrared measures of hydrogen adsorbed in front of Zn^4+^ or O^2−^ sites of MOF frameworks as well as REMPI measures on solid water at very low temperatures. The jump from the solid to the molecule, **UY**, was found efficient in a different context of noble metals [28]. But the metallic case is quite different, because the electron excitations inside the conduction band might be infinitesimal. The reverse jump from the molecule to the solid: **VY** was suggested once by S. Sugano in the case of chromia catalysts (S. Sugano 1987, personal communication), but astonishingly never published. As a possible example of application, hydrogen in silicon was shown to transfer a slight electron charge to the solid [38]. Note that exchange and transfers might mix their effects or one of these overcomes the others. In the above definition of the repulsion–contact tensor: ${\left\{\text{C}.\text{y}\right\}}^{1}$, the exchange and molecule → solid transfers have a different sign from the solid → molecule ones. Attractive and repulsive *o*–*p* interactions have identical rates. The concept of molecule–solid electron attraction seems meaningful for the *X* and *V* processes, because the solid can manage more easily an extra electron than the molecule that has a strong internal repulsion, but conversion is a dynamical phenomenon also favoured by repulsions.

### 4.3. Nuclear conversion

Consider now the low-frequency nuclear ‘ortho–para’ response, denoted spin–orbit-Coulomb-hyperfine contact process: SOCY = SOXY + SOUY + SOVY, resulting from the coordinate action of three couplings included in the perturbation Hamiltonian

$$\text{H}-{\text{H}}_{\text{0}}=\Lambda +\text{C}+\text{Y}.$$

The observed nuclear transitions are real and result in energy and momenta exchanges between the molecule and the solid. Contradistinctly, the electron mixings are described as closed loops in the electron orbital space. These channels rely on a small breaking of the non-magnetic symmetry by non-diagonal ‘bonding–antibonding’ interactions. The singlet–triplet mixings include electron repulsion, hyperfine contacts, SO as well as spin-other orbits and mutual exchange interactions, but the electron average of the electronuclear operator **O** is performed inside the fundamental ground state *G*

$$|\text{O}(j_{p};j_{o})|{\;}^{2}=\sum_{m_{p},m_{o},i_{o}}|G;p|\text{O}|G;o|{\;}^{2}.$$

The conversion process is then induced by purely nuclear transitions, and the spectral densities are expressed at the *o*–*p* energies $\varepsilon_{op}=h\nu_{op}=\varepsilon_{j_{o}}-\varepsilon_{j_{p}}$.

$$W_{n}(j_{o},j_{p})=\sum_{m_{op},i_{0}}W_{n}(j_{o},j_{p},m_{op},i_{0})=|\text{O}(j_{p};j_{o})|{\;}^{2}J(\varepsilon_{op}).$$

The conversion operator is constructed through a third-order combined action of the previous **T** operator and the SO Λ. **T** induces a double and simultaneous singlet–triplet (*S*−*T*) transition [nuclear spin (Δ*I* = 1) and electron spin (Δ*S* = 1)], whereas Λ performs only the electronic one. At third order, the perturbation combines three virtual steps, and there are six of such terms: $\Lambda .\text{C}.\text{Y}+\Lambda .\text{Y}.\text{C}+\text{Y}.\Lambda .\text{C}+{\text{[ ]}}^{†},$ leading to an effective electronuclear Hamiltonian:

$$\text{O}=[\Lambda .{\text{G}}_{1}.\text{C}.{\text{G}}_{1}.\text{Y}+\Lambda .{\text{G}}_{1}.\text{Y}.{\text{G}}_{0}.\text{C}+\text{Y}.{\text{G}}_{1}.\Lambda .{\text{G}}_{0}.\text{C}]+[\;]{\;}^{†},$$

where the Green operators ${\text{G}}_{S}$ have been defined previously for the singlet and triplet channels: *S *= 0 or 1. The global electron canonical average, performed inside the fundamental ground state *G*, ⟨G|O|G⟩ is a nuclear operator

$$\langle G|\text{O}|G\rangle =\langle {\;}^{1}G|\Lambda .{\text{G}}_{1}.\{\text{C}{\text{G}}_{1}\text{Y}+\text{Y}{\text{G}}_{0}\text{C}\}|{\;}^{1}G\rangle .$$

The operator $\text{Y}.G_{1}.\Lambda .G_{0}.C$ (and its conjugate) here omitted will be incorporated as a correction of the main part (see electronic supplementary material, A3.2). The first Green projector **G**_1_ accounts for the spin triplet excitations ${\;}^{3}\epsilon (\xi \eta )$, whereas the next ${\text{G}}_{1}$ and ${\text{G}}_{0}$ collect the triplet ${\;}_{\;}^{3}\Gamma$ and singlet ${\;}_{\;}^{1}\Gamma$ channels

$$\langle {\;}^{1}G|\Lambda |{\;}^{3}\epsilon \rangle \sum_{\Gamma }\frac{\left\{\langle {\;}^{3}\epsilon |\text{C}|{\;}^{3}\Gamma \rangle \langle {\;}^{3}\Gamma |\text{Y}|{\;}^{1}G)\rangle +\langle {\;}^{3}\epsilon |\text{Y}|{\;}^{1}\Gamma \rangle \langle {\;}^{1}\Gamma |\text{C}|{\;}^{1}G\rangle \right\}}{\varepsilon_{\Gamma }}.$$

Inserting the various matrix elements expressed in electronic supplementary material, tables A3–5, the orbital average of the electronuclear operator is obtained as series of products of three scalar products

$$\langle G|\text{O}|G\rangle =\sum_{\xi \eta }\left[{\text{Y}}^{1}(\hat{ab}).\{\text{C}.\text{y}\}{\;}^{1}(\xi \eta )][{\text{i}}^{1}.\;\sigma^{1}][{\left\{\lambda \right\}}^{1}(\xi \eta )\;.\;\sigma^{1}\right].$$

**Figure 3.**
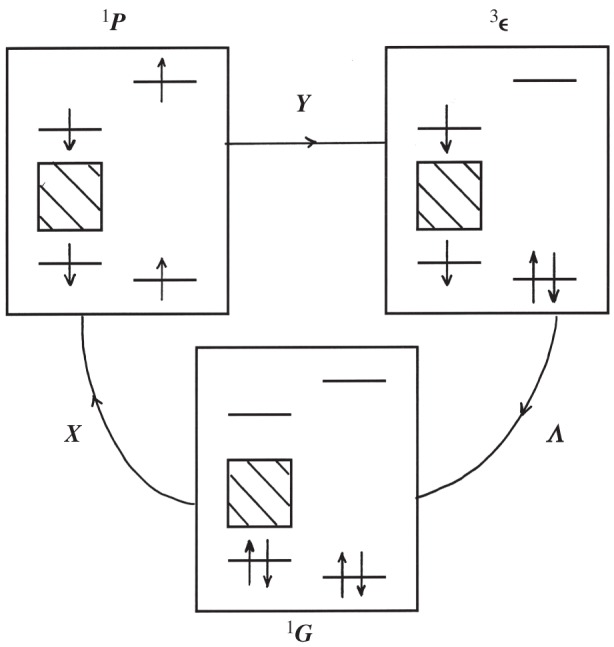
A singlet channel: the molecule–solid electron repulsion **C **= **X** excites simultaneously the molecule and the solid antibonding states (a two-electron excitation). In such a neutral state, the bonding (and antibonding) electrons have opposite spins and the molecule rotates differently (not represented). In the next step, the molecular electrons transfer their spin momenta to the nuclei by hyperfine contact **Y**. For appreciable **SO** coupling (Λ) in the solid and sufficient scattering time, the solid relaxes before the molecule leaves. (Only a nuclear energy is exchanged with the solid.) If the **SO** coupling is weak or the collision too fast, an exciton is emitted, and propagates before mutual annihilation.

**Figure 4.**
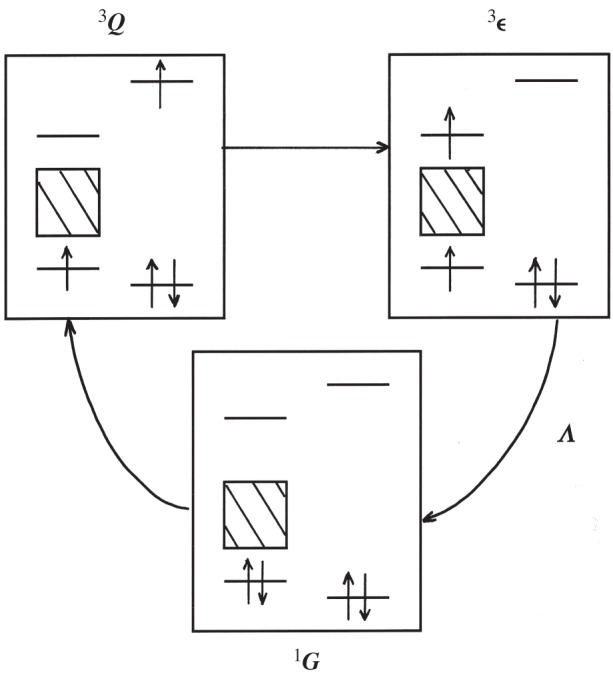
A triplet channel: an example of a solid valence electron jumping on the molecular u state by hyperfine contact (a single-electron excitation). The shifted electron and its remaining pair in the solid build a magnetic electronic state that flips the nuclear spins. The electron repulsion brings back the shifted electron to the solid through the molecule–solid bridge (the bonding of the antibonding states) emitting (or not) an exciton. The tunnelling is denoted as one **UY** channel (or **SOUY** for sufficient **SO**).

It is possible to uncouple and recouple the electron and nuclear tensors to obtain a compact form, separating the solid and molecule tensors, as expressed in A3.2 in electronic supplementary material [40,41]. However, it is as simple to express the nuclear matrix elements directly by performing the electron spin summation (electronic supplementary material, A1.2) and the ground state average O(p;o)=⟨G;p|O|G;o⟩, to obtain the link between the ortho–para manifolds: $\left(p;o)=(j_{p},m_{p};j_{o},m_{o},i_{o}\right)$:

$$\text{O}(p;o)=a_{op}\sum_{\xi \eta }\left[{\left\{\lambda \right\}}_{i_{o}}^{1}.{\left\{\text{C}.\text{y}\right\}}_{m_{op}}^{1}](\xi \eta \right),$$

as an expansion over all excitons (ξη). Each *o*–*p* transition operator matrix element is thus written as a product {λ}{Cy}={λ.C.y} of non-diagonal Coulomb integrals *C*, weighted by magnetic SO and contact amplitudes: λ∼{Λ/ε} and y∼{Y/E}. They represent effective electromagnetic energies either repulsive or attractive between the electrons of the solid and the molecular ones (linked to their protons). The angular momentum transfer is the most efficient for the antibonding electron orbitals: molecular ones *u* pointing in the direction of the solid, or solid ones *η* towards the molecule. Figures 3 and 4 depict two possible channels. Figure 3 represents one spin-singlet path. The first X-step illustrates how the purely electrostatic Coulomb interaction is able to change the electron spins of the molecule and the solid (the total spin remaining conserved). (The consequent rotation slip *J *= 1 → *J *= 0 is not indicated.) The second *Y*-step transfers the molecular electron spin to the nuclear spins. Finally, the last SO-step relaxes the solid excitation. One triplet path is represented in figure 4 where a valence electron jumps on the molecule by a first hyperfine step *Y*, introducing an electron spin inside the molecule which ‘flips’ the protons. A second Coulomb repulsion-step brings the excited molecular electron back to the conduction band of the solid, and changes simultaneously the rotational momentum. For both channels, the last SO step reintegrates the excited electron in the valence band. Every step induces the breaking of a different selection rule and affects the partial angular momentum conservation: Λ changes the solid electron spin, **C** the molecular rotation *J* and the solid angular one, **Y** the electronic and the nuclear spins *S* and *I*. The bonding characters are also changed in the transient states. Altogether, the nuclear *o*–*p* transition probabilities take the simple form

$$W_{n}(j_{o},j_{p})=a_{op}^{2}\;J(\varepsilon_{op})\sum_{\xi \eta }|C_{n}(\xi \eta )|{\;}^{2},$$

where the ortho–para electromagnetic interaction $C_{n}(\xi \eta )$ is now being defined by

$$|C_{n}(\xi \eta )|{\;}^{2}={\left\{\lambda .\text{C}.\text{y}\right\}}^{2}(\xi \eta ).$$

The effective singlet–triplet excitonic mixing is obtained as follows

$${\left\{\lambda .\text{C}.\text{y}\right\}}^{2}(\xi \eta )=\frac{\sum_{\mu ,\nu =x,y,z}|\Lambda_{\mu }{\left(\text{C}.\text{y}\right)}_{\nu }|{\;}^{2}(\xi \eta )}{2{\left(\varepsilon_{\xi \eta }\right)}^{2}},$$

(the C.y(ξη) are defined in 3*b*). A different version of the nuclear conversion was originally suggested by Sugimoto & Fukutani [23]. In that channel denoted **SOPY**, the surface electric field polarizes the hydrogen and introduces some orbital momentum inside the molecular space. The SO interaction transfers that momentum to the electron spins, then able to contact their protons. The resulting rate, enhanced by a level crossing and strong surface electric gradients, has been detailed along the electronuclear path suggested by their authors [33]. In the present formalism, the conversion rate relative to the molecular SO interaction is expressed differently (see electronic supplementary material, A3.2).

## 5. Electromagnetic channels and materials

First observe that almost all existing theories of hydrogen conversion consider a single and discrete catalyst state. Astonishingly, the role of a continuous series of states, electron bands, on the conversion of hydrogen interacting with a solid, has never been studied sufficiently. At one exception, the silver metallic case was interpreted on the basis of a (111) surface state and a short portion of the surface band around the Fermi level was taken into account [28] (an early oversimplified calculation failed to obtain the observed orders of magnitude [43]). For the fast metallic rates, there is no distinction between the electron and nuclear families, because the most important electron excitations involve nuclear *o*–*p* energies. For insulators, there is a threshold, and the exciton energy necessary to reach the triplets $\varepsilon_{\xi \eta }$ must at least be equal to the gap Δ. A large part of the following discussion is thus devoted to the influence of the width and position of that gap on the conversion processes.

Let us recall the characteristic conversion times observed with different hydrogen–solid configurations. For noble metals, it took about 1–20 min depending upon the surface orientations to register an appreciable change in the spin isomer ratio. Distinctly hydrogen conversion inside silicon samples needed between 8 and 230 h, owing to the samples and experimental temperatures. Observations on ionic samples such as Zn-MOF and solid water systems, characterized by non-saturated bonds and giant surface electric fields, necessitate a few minutes, similar to the metallic case, whereas the results observed on covalent insulators approach the semiconductor times of many hours [33].

Two distinct time scales: (1–10 min) and (1–100 h) thus stand out, and it is desirable to relate these time scales to the different channels and solid properties. Each channel is a reaction path ‘℘*’* either nuclear (*n*) or electronuclear path (e), defined by the nature of the exciton involved (ξ−η), by a virtual ‘molecule–solid’ charge-fluctuation (P,Q,R)≡(X,U,V) and a couple of ortho and para states. The parameter space can thus be summarized as

$$℘\equiv \{(e,n),(\xi ,\eta ),(X,U,V)\}\{j_{p},m_{p};\;j_{o},m_{o}\}.$$

The global conversion rate, inversely proportional to the measured conversion time, sums all different channels, assumed independent one from another

$$W_{op}=\sum_{℘}W_{℘}=a_{op}^{2}\sum_{℘}C_{℘}^{2}\;J(\omega_{℘}).$$

${\text{C}}_{℘}$ represents the effective ‘repulsion–contact’ couplings that have the dimension of a frequency, the numbers $a_{op}$ are rotational matrix element functions of the ortho- and para-connected manifolds. $J(\omega_{℘})$ are spectral densities at the transition frequencies $\omega_{℘}$ of the paths, homogeneous to a time. To compare the different conversion channels, the following discussion considers: (i) the relative influence of the electron and nuclear paths, (ii) the ‘excitonic’ extension outside the solid, (iii) the transients (*P*, *Q*, *R*), their corresponding couplings (X,U,V) and excitation energies, and (iv) the distinction between discrete levels and continuous bands owing to the interacting solid catalyst.

### 5.1. Electronuclear and nuclear paths

Approximate expressions will be used to consider orders of magnitude and relate the theory to the experimental measures. The molecule–surface distance will be assumed a random variable of the thermostat where molecular vibrations, phonons, ad- and desorption exchanges participate incoherently [38]. The functions of the distance will be assumed to obey a Markov process with exponential correlation functions: *f*(*t*) = exp−*t*/*τ* decaying with a characteristic time *τ*. Consequently, the spectral densities are of the Lorentzian form: *J*(*ω*) = 2*τ*/1+(*ωτ*)^2^. Usual scattering times are of the order: *τ*_≈_ 10^−12^ to 10^−13^ s. Owing to the electronic or electronuclear nature of the transition, two different dynamical responses might occur (i) for electronic conversion involving energies $\hbar \omega_{e}$ larger than the eV: $\omega_{e}\tau \gg 1$ and thus: $J_{\tau }(\omega_{e})\approx 2\text{/}\tau \omega_{e}^{2}\ll \tau$; (ii) differently, for the nuclear *o*–*p* transitions of the order of ℏ*ω_n _*≈ 10–30 meV: $\omega_{n}\tau \approx 1$ and $J_{\tau }(\omega_{n})\approx \tau$. Comparing the two processes for the main 0–1 transition $(\omega_{n}=\omega_{01}),\tau \approx \omega_{01}^{-1}\;\approx 3\times 10^{-13}\;\text{s}$, the nuclear process has a much faster response than an electronic one of about 1 $\text{eV:}\;J_{\tau }(\omega_{01})\text{/}J_{\tau }(\hbar \omega_{e}=1\;\text{eV})\approx 10^{+4}$. It might, thus, be tempting to attribute the fast *o*–*p* rates (1–10 min) to the nuclear paths, which would be misleading, because other factors compete. Let us compare them more carefully. The minus square of the exciton energy $\varepsilon_{\xi \eta }$ appears both in the electronic spectral density and in the singlet–triplet admixture of the nuclear SOPY path. Both CY and SOCY transition rates are $∼{\varepsilon_{\xi \eta }}^{-2}$, and consequently any (*e*) or (*n*) conversion rate can be expressed as

$$W_{℘}=\sum_{\xi }F_{℘}(\xi \eta )E_{℘}^{-2}\varepsilon_{\xi \eta }^{-2}.$$

$E_{℘}$ appears as an energy barrier to be overcome by the different paths $℘:E_{X}=\varepsilon_{gu},E_{U}=\varepsilon_{\xi u}$, $E_{V}=\varepsilon_{g\eta }$ or $\varepsilon_{\xi \eta }$. For each electronic CY path of exciton energy $\varepsilon_{\xi \eta }$ relative to a channel ℘=X or *U* or *V*, the electronuclear rate has the following form: $W_{e}(\xi )\approx |{\left(\text{CY}\right)}_{℘}\text{/}E_{℘}\varepsilon_{\xi \eta }|{\;}^{2}\tau^{-1}$, whereas for the nuclear SOCY path relative to the same channel ℘, it writes: $W_{n}(\xi )\approx |{\left(\text{CY}\right)}_{℘}\Lambda /hE_{℘}\varepsilon_{\xi \eta }|{\;}^{2}\tau$. The term ${\left(\text{CY}\right)}_{℘}\text{/}E_{℘}\varepsilon_{\xi \eta }$ is common to both (*e*) and (*n*) processes and thus by defining an SO period by $T_{℘}=h/\Lambda (\xi \eta \text{)}$, the ratio of the (*e*) and (*n*) conversion rates is about

$${\left(\frac{W_{e}}{W_{n}}\right)}_{℘}\approx {\left(\frac{T_{℘}}{\tau }\right)}^{2}.$$

$T_{℘}$ represents an average period to establish a local electron spin singlet–triplet admixture and τ the dynamical scattering time. For $\tau \gg T_{℘}$, appreciable singlet–triplet coupling or soft collisions, the nuclear conversion becomes the leading process. For instance, if *τ *≈ 10^−12 ^s, then $\Lambda \gg 30\;\text{c}{\text{m}}^{-1}$ is necessary. On the contrary, the electron process overcomes the nuclear one for hard collisions: $\tau \ll T_{℘}$ (for instance, if *τ* _≈ _10^−13 ^s, $\Lambda \ll 300\;\text{c}{\text{m}}^{-1}$ is often satisfied). In other words, the interaction time is too short for appreciable SO-induced conversion in the near vicinity of the molecular scattering, and the exciton propagates. In between when $\tau \approx T_{℘},$ both channels contribute to the conversion process.

### 5.2. ‘Exciton’ extension outside the solid

**Figure 5.**
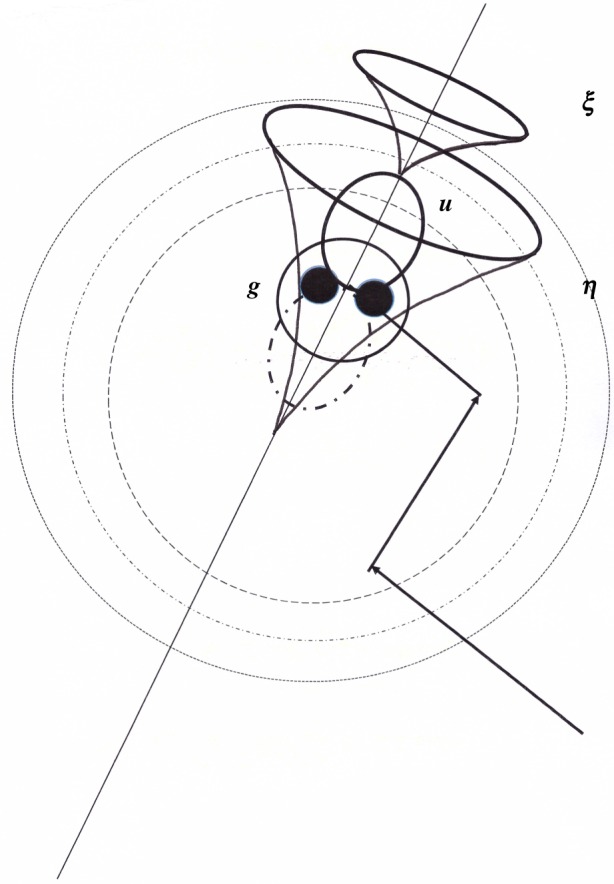
The scattering. One hydrogen molecule, whose centre **m** follows a fast and erratic random walk inside a solid potential well, is represented at a moment when the molecule scatters the solid. The molecule is represented by the two protons and its internuclear axis. The two molecular electrons are distributed in the bonding and almost spherical **g**(1 s) state. Dotted circles figure the solid well equipotentials. The empty molecular antibonding **u**(1 s) state, figured by an ellipse, approaches higher potentials (repulsive hard wall). A pair of conduction η and valence ξ state exponential tails of axial symmetry are directed towards the molecule. The excited state η has a much larger extension and overlaps appreciably with the molecular antibonding state.

The hydrogen molecule is represented in figure 5 in its erratic motion when colliding with a solid potential wall. The molecule bonding electrons are spherically distributed around the centre of mass m, while the antibonding orbital *u* is assumed to be axially spread along a symmetry axis. Also figured are valence ξ and conduction *η* wave function tails, which are exponential far outside the solid. In the following we denote these various tails for (ξ,η) states by ${\text{e}}^{-\xi r}$ and ${\text{e}}^{-\eta r}$, whereas the molecular ones g and u in the solid space behave as ${\text{e}}^{-\lambda_{g}r}$ and ${\text{e}}^{-\lambda_{u}r}$. The best HF hydrogen orbital g is obtained for *λ_*g*_*=1.189 arb. units [44], whereas *λ_*u*_* is the function of the bound state mixture. The CY tails of the XY, UY and VY channels are figured in electronic supplementary material, table A8. The Coulomb–contact terms have their main amplitudes, either in the solid space or in the molecular one. The spatial decrease of the ‘repulsion–contact’ interaction in molecular space arises from the inverse sum of the solid tails: ${\left(\xi +\eta \right)}^{-1}$, whereas in the solid space, it can be either a purely molecular decrease ${\left(\lambda_{g}+\lambda_{u}\right)}^{-1}$ or a mixed one ${\left(\lambda_{u}+\eta \right)}^{-1}$. Usually, the bonding states are more localized than the antibonding ones: $\lambda_{g}\ll \xi \ll \eta ,\lambda_{u}$. Therefore, the repulsion between a valence electron and a molecular ‘*a*−*b*’ one, conjugated to the contact of a conduction electron with the H_2_ nuclei: ⟨ξξ||ξu⟩η(m) is predominant in the UY channels. It competes with the exchange integrals ${\text{X}}_{\xi \eta }$ between two molecule and two solid mixed densities, bonding and antibonding (*b*−*ab*): $\left\langle \xi \eta |1\text{/}r_{12}|ug\right\rangle$. Thus, the relative surface spatial extensions allow a first and partial classification:

(a) λ≫∼ξ. When the conduction and valence electrons have a larger extension than the molecular ones, the main repulsion occurs within the molecular space. Ionic insulators with small affinities and ionization potentials favour charge-transfer processes. It seems also to correspond to the case of noble metals Ag, Cu [27] where (η∼ξ) belong to the same conduction band and thus a global soft decrease $∼{\text{e}}^{-2\eta d}$.
(b) $\xi \gg \lambda_{u}>\eta$: for insulators of small gap, and semiconductors of low work functions, when the conduction states are more extended than the molecular ones, but not the valence states, the major attraction/repulsion occurs in solid space as $∼{\text{e}}^{-(\lambda_{u}+\eta )r}$. Charge-transfer processes compete with the exchange one.
(c) $\xi >\eta >\lambda_{u}$: for insulators of large gap, valence states and even the conduction states are more localized, at least more than the molecular ‘*a*−*b*’ one. Main repulsion in solid space decreases as $∼{\text{e}}^{-(\lambda_{g}+\lambda_{u})r}$ which favours the exchange process.

### 5.3. ‘Repulsion–contact’ couplings and excitation energies

The solid and molecular electron antibondings at the solid surfaces provide efficient paths for the electron tunnelling to reach the protons and convert the hydrogen to thermal equilibrium proportions. It is worthwhile to describe a little further the general characters of the neutral **XY** and charge-transfer **UY** and **VY** processes and compare their amplitudes. More important than the common rate dependence on the exciton energy: $∼{\varepsilon_{\xi \eta }}^{-2}$, the rate dependence $∼E_{℘}^{-2}$ on the individual channel barriers $E_{℘}$ characterizes both the (*e*) and (*n*) families. The paths through different charge repartitions: *P*, *Q* or *R* necessitate to overcome different energy barriers: $E_{X}=\varepsilon_{gu},E_{U}=\varepsilon_{\xi u},E_{V}=\varepsilon_{g\eta }$ or $\varepsilon_{\xi \eta }$, for the hydrogen conversion to occur (compare, for example, figures 3 and 4). These excitation energies are the main parameters that modulate the electrostatic links. Quite generally: $\varepsilon_{gu}>\varepsilon_{\xi u}>\varepsilon_{g\eta }>\varepsilon_{\xi \eta }$. Two figures are important: the weak solid ‘excitonic’ energy $\varepsilon_{\xi \eta }$ close to the gap value ≈ *Δ* and the large molecular one $\varepsilon_{gu}$ close to the hydrogen ionization potential. The hydrogen fundamental bonding orbital lies deep in most cases inside the valence band, at $\varepsilon_{g}\approx -15,8\;\text{eV}$. Adding another electron in the ab *u* state costs $\varepsilon_{gu}$ (see electronic supplementary material, table A2) if the final compound is the triplet ^3^*Σ*_u_. The ‘*a*–*b*’ molecular excited state $\varepsilon_{u}$ is a resonance in the gas, but becomes almost a bound state in the near vicinity of the solid. On the one hand, it remains linked to plane waves for physisorbed molecules. On the other hand, it is delocalized and broadened by the bonding with the conduction states as represented in figure 1. $\varepsilon_{u}$ is thus pushed downwards and appears as a key parameter distributed around 5–10 eV. $\varepsilon_{\xi u}$ locates it with respect to the valence band and represents the energy necessary to transfer one solid electron to the molecule excited state close to the vacuum level. For an electron state arising from the top of the VB: $\varepsilon_{\xi u}\approx \Phi$ is the work function of the solid in the presence of a hydrogen molecule. $\varepsilon_{g\eta }$ locates the conduction band with respect to the molecular bonding *g* (figure 1). It is the energy received by the system when a conduction electron jumps on the ionized hydrogen ground state. If the electron was around the bottom of the conduction band, then it corresponds to an affinity energy of an excited molecule in the presence of a solid: $\varepsilon_{g\eta }\approx \text{A}$. These four parameters are related by: $\varepsilon_{g\eta }+\varepsilon_{\xi u}=\varepsilon_{\xi \eta }+\varepsilon_{gu}$ and particularly: $A+\Phi =\Delta +\varepsilon_{gu}$.

Average Coulomb repulsion, hyperfine contact, ionization and gap energies, respectively denoted *C*, *Y*, *Φ* and Δ, are now considered. The electronic conversion time $t_{op}^{\left(e\right)}$ has the following order of magnitude

$$\frac{t_{op}^{\left(e\right)}}{\tau }\approx {\left(\frac{\Phi \Delta }{C\;Y}\right)}^{2}\;\text{.}$$

With Φ≈10 eV, $Y\approx 3\times 10^{-6}\;\text{eV}$ and $\tau \approx 10^{-13}\;s$, the conversion time expressed in seconds reduces to $t_{op}^{\left(e\right)}\approx {\left(\Delta /C\right)}^{2}$. If both the gap and repulsion energies are weak (*C* ≈ 10 meV and Δ ≈ 2 eV), then the process will need around 10 h. For similar gap and stronger repulsion (*C* ≈ 60 meV), the process is faster around 15 min. On the contrary, for a larger gap of 10 eV (and weak repulsion: C ≈ 10 meV), the conversion is much slower, around 250 h. As already noted, these rates might also be speeded by strong SO interactions. These figures, close to the experimental observations, display the extreme sensitivity of the measured conversion rates to the electronic parameters of the solid.

### 5.4. Discrete levels and bands

**Figure 6.**
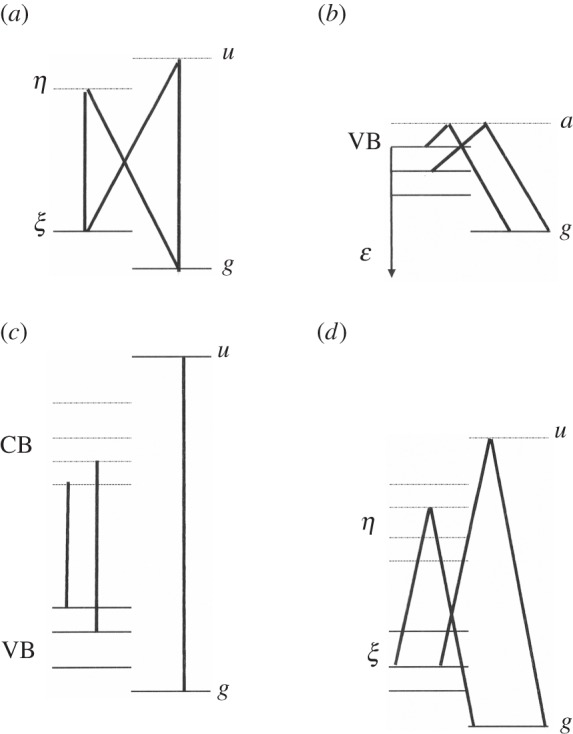
Molecule–solid electron couplings in different configurations: (*a*) Mixing between solid and molecule discrete states. (*b*) Mixing between discrete and band states illustrated by a ‘p’-type semiconductor with acceptor level ‘a’ (Main charge transfer towards the solid). For n-type semiconductors with a donor state ‘d’ charge, the transfer arises towards the molecule. (*c*) ‘Valence band → conduction band’ mixings in the case of a large gap insulator. Main exchange tunnelling sums all possible VB and CB pair states, and involves a double band summation including their respective density of states. (*d*) For a small gap semiconductor, the charge-transfer tunnelling not only overcomes the exchange ones, but also involves a double band summation.

Let us now return to a qualitative discussion and illustrate how the summation over the valence ξ and conduction *η* states influences the conversion rate by considering a few types of non-magnetic solids, as represented in figure 6.

1. First, *an ionic compound* where the highest occupied orbital ξ is separated from the first empty excited state *η* by a large excitation energy Δ, assuming that other ones are farther apart (figure 6*a*). Then, the exchange process is the fastest, because the internal contact is much larger than the solid one: Y(gu)≫Y(gη), and the conversion rate contains a single term
  $$W=W_{X}∼\frac{F_{X}}{\Delta^{2}{\left(\varepsilon_{gu}\right)}^{2}}$$
  This was the model investigated to discuss the case of conversion observed above Zn^3+^ cations in MOF 74, although it was also shown that covalent bonds multiply the number of available channels, resulting in a faster conversion. If the gap is small and the work function large, then the molecule bonding energy being close to the valence band maximum, the charge transfer ‘V’ towards the solid becomes dominant. If, in addition, a few impurity discrete levels lie in the gap, the conversion rate is written as a discrete sum.
2. *For n type semiconductors* where the donor state ‘d’ is localized near the conduction band, the conversion is favoured by the U process. In that case, an ‘impurity’ electron ξ might jump over the molecular antibonding u orbital directed towards the solid, and thus ‘alloyed’ to the conduction states. Such a configuration is an example of a transition ‘initial discrete → excited band’. The summation over the band states is conveniently converted into a continuous integration including the CB density of states. Because the coupling matrix elements are dependent weakly on the eigen-energies, the form factor $F_{U}$ can be averaged over the CB: $\left\langle F_{U}\right\rangle$ and extracted from the integral
  $$W_{U}∼\frac{\left\langle F_{U}\right\rangle }{{\left(\varepsilon_{d}+\varepsilon_{u}\right)}^{2}}\int_{\mathrm{CB}}\frac{n(\varepsilon )\;\text{d}\varepsilon }{{\left(\varepsilon_{d}+\varepsilon \right)}^{2}}.$$
  All energies are positive and measured from the bottom of the conduction band: $\varepsilon_{\xi }=\varepsilon_{d}+\varepsilon$ and $\varepsilon_{\xi u}=\varepsilon_{d}+\varepsilon_{u}$. Similarly, f*or p type semiconductors,* the transition ‘initial band → discrete excited’ is illustrated in figure 6*b* by an acceptor level ‘a’ slightly above the VB. Main charge transfer then arises towards the solid and with a similar approximate VB average ⟨*F_V_*⟩
  $$W_{V}∼\frac{\left\langle F_{V}\right\rangle }{{\left(\varepsilon_{a}+\varepsilon_{g}\right)}^{2}}\underset{\mathrm{VB}}{\int }\frac{n(\varepsilon )\;\text{d}\varepsilon }{{\left(\varepsilon_{a}+\varepsilon \right)}^{2}},$$
  (all positive energies measured from the top of the valence band: $\varepsilon_{\xi \eta }=\varepsilon_{a}+\varepsilon$ and $\varepsilon_{g\eta }=\varepsilon_{a}+\varepsilon_{g}$).
3. Now let us consider an ensemble of ‘VB Band →CB Band’ transitions where, in the case of an *insulator with large band gap* comparable to $\varepsilon_{gu}$, the exchange tunnelling is the major one despite slightly larger charge-transfer repulsions. It sums all possible transitions between the valence and conduction band, as represented in figure 6*c*, leading to a probability that involves a double integration over the CB and the VB
  $$W_{X}∼\frac{\left\langle F_{X}\right\rangle }{{\left(\varepsilon_{gu}\right)}^{2}}\int_{\mathrm{CB}}\int_{\mathrm{VB}}\frac{n(\varepsilon_{c})n(\varepsilon_{v})\;\text{d}\varepsilon_{v}\;\text{d}\varepsilon_{c}}{{\left(\Delta +\varepsilon_{c}+\varepsilon_{v}\right)}^{2}}.$$
  The energy $\varepsilon_{c}$ is here referred to CB bottom and $\varepsilon_{v}$ to the VB top.
4. For *an intrinsic semiconductor of small gap*, the charge-transfer tunnellings overcome the exchange ones
  $$W_{U}+W_{V}∼\int_{\mathrm{VB}}\int_{\mathrm{CB}}\frac{n(\varepsilon_{v})\;\text{d}\varepsilon_{v}n(\varepsilon_{c})\;\text{d}\varepsilon }{{\left(\Delta +\varepsilon_{c}+\varepsilon_{v}\right)}^{2}}\left\{\frac{F_{U}}{{\left(\Phi +\varepsilon_{v}\right)}^{2}}+\frac{F_{V}}{{\left(A+\varepsilon_{c}\right)}^{2}}\right\},$$
  where Φ denotes the position of the *u* ab-state (close to the vacuum) referred to the top of the VB (the energy necessary to extract one solid electron and locate it on the molecular ‘*a*−*b*’ state) and A the position of the *g* state referred to the bottom of the CB (the energy necessary to extract one molecular electron and delocalize it in the conduction band).

Summarizing, our model stresses the main influence of the solid and molecule on respective electronic eigen-energies. The position of the molecular antibonding level with respect to the conduction band is determinant to build a kind of impurity bound state at the surface, partly delocalized by incorporating a number of conduction states. Although it remains empty in the physisorption regime, it acts as a molecule–solid bridge through which the conversion might flow. In particular, when the solid energies (gap Δ and ionization potential Φ) are small, the electron transfer from the valence band to the molecular antibonding u-state is the fastest conversion path. Such **UY** processes have channels $∼(\varepsilon_{\xi u}\varepsilon_{\xi \eta }){\;}^{-2}$. Most of the interband transitions $\varepsilon_{\xi \eta }$ have energies larger than the gap Δ, because the states densities are low at the bands extrema. Differently, the effective ionization potential $\varepsilon_{\xi u}$ to extract an electron from the solid and locate it on the molecule (on the bridge) is smaller than the work function Φ for complete delocalization. The approximation $\varepsilon_{\xi u}\varepsilon_{\xi \eta }\approx \Phi \Delta$ in the integration average appears, therefore, justified. But in the other case, when the solid work function, Φ is large, but the gap Δ and affinity A (of the ion ${\text{H}}_{2}^{+}$) both small, and a molecular electron jumps more easily in the solid conduction band. Such **VY** processes have channels whose rates are $∼(\varepsilon_{g\eta }\varepsilon_{\xi \eta }){\;}^{-2}$. Again, $\varepsilon_{\xi \eta }$ is larger than the gap Δ and again differently, the energy to extract an electron from the molecule and delocalize it in the solid conduction band: $\varepsilon_{g\eta }$ is smaller than the molecule ionization potential I. The approximation $\varepsilon_{g\eta }\varepsilon_{\xi \eta }\approx I\Delta$ appears, in that case, convenient to describe these **VY** channels. Finally, when the gap is still small but located in the middle of the (*b*−*ab*) and (*g*−*u*) molecular gap: $A\approx \Phi ,\varepsilon_{gu}\approx A+\Phi$, the exchange **XY** rate is favoured. Its energy barrier factor ($∼\Delta^{-2}{\left(A+\Phi \right)}^{-2}$) is similar to the charge-transfer ones, but the hyperfine contact through the molecular one (between the protons and the 1 s electrons) is stronger. For larger gaps, all processes have decreasing rates: as $∼\Delta^{-2}$ for low gaps and as $∼\Delta^{-4}$ for larger ones.

## 6. Concluding comments

Models have here been provided to interpret new experiments on the ortho–para conversion of hydrogen, either ‘physisorbed’ at the surface or diluted inside non-magnetic solids. Optical methods (Raman, infrared or REMPI) and electronic ones (EELS, STM) are now sufficiently precise to distinguish the ortho and para lines, disentangle the rotational properties from the vibronic ones and measure their slow relaxation patterns.

It is clear now that the excited states of the molecule and the solid provide efficient reaction paths to convert hydrogen molecules interacting with non-magnetic solids in time scales larger than one or a few minutes (conversion times by permanent moments are shorter than a fraction of a second). The conceptual origin of the interpretation relies on the magnetic excitations of non-magnetic catalysts (and of the hydrogen molecule, as well). These electron excitations of triplet spins use the molecule and solid antibonding states of appreciable surface extension. The admixture of the antibonding molecular state with the conduction band allows a partial delocalization of a molecular electron in the solid and inversely a partial localization of a conduction electron on the molecular edge. The ‘bridge’ facilitates the hyperfine contact of the electrons with the hydrogen protons. The repulsion performs the molecular rotation transition by the excitation of higher orbital momenta states, whereas the consequent electron delocalization enhances their contact with the nuclear spins.

The described model enlarges previous theoretical studies by extending the electron basis to charge-transfer states and ‘continui’ of band states. Three main ‘symmetry-breaking’ interactions have been combined to build a conversion operator. The molecule–solid electron repulsion **C** breaks the molecule inversion symmetry and mixes ‘gerade’ (*g*) and ‘ungerade’ (*u*) states, which are respectively bonding (*b*) and antibonding (*ab*). The magnetic hyperfine contact **Y** breaks both the electron and nuclear spin angular momentum conservation: Δ*S* = Δ*I* = 1. A general singlet/triplet mixing collects one- and two-electron SO (and exchange) interactions, breaking the solid or the molecule electron spin conservation. The electron system acts as a key that regulates the thermal flow between the molecule and the solid. The various conversion channels appear, thus, as reaction paths tunnelling through transient states, branches or loops in the electronic space that must overcome different energy barriers to complete the molecule rotational and nuclear spin transitions. The operatorial formulation of the conversion process in terms of Green projectors over the complete excited spectra, as described in the first three sections, is exact. However, in the fourth section, the limited electron basis and the exponential correlation model used to describe the solid–molecule distance are approximations that allow a qualitative analysis in terms of a few parameters. The electron repulsion and SO interaction remain parameters that characterize each solid and particular scattering geometry (the intramolecular hyperfine contact can be calculated independently). Other parameters are energetic and depend upon the relative position of the solid bands and gap compared with the molecular bonding and antibonding levels, as represented in figure 1. Work function, first ionization and affinity levels are convenient parameters to discuss the conversion rates and relate them to particular solid–molecule scatterings.

Each channel is a reaction path ‘℘*’* that corresponds to a tunnelling through a particular barrier. The first family, denoted electronic (e) or Coulomb–contact **CY**, considers the electrons and nuclei as interdependent systems. A spin-triplet exciton is emitted in the solid, by a transfer of a valence electron to the conduction band, and travels around until annihilation. The emission might be achieved by electron exchanges **C = X**, or electron transfer towards the molecule **C = U**, or towards the solid **C = V**. Each transfer is complemented by a different contact **Y** between the electrons and the nuclei. Because the nuclear and electron angular momenta (and energy) transfers are simultaneous, the conversion occurs at the high frequencies of several eV.

The second family of processes, denoted nuclear (*n*) or spin–orbit-coulomb–contact (**SOCY)**, considers that the preceding **CY** path leaves the system in a transient virtual state and complements it by an additional **SO** one, which brings back the electrons in their original ground state. The net transition is then purely nuclear, and conversion occurs at the low frequencies of several meV. An approximate criterion is suggested to decide which one of these two families is the most efficient for the hydrogen conversion on insulators and semiconductors. By defining a singlet/triplet characteristic time *T*, as h times the inverse of the non-diagonal SO frequency and comparing it to the correlation time τ (roughly the scattering time), two different dynamical regimes are distinguished. If T≫τ, which occurs for short elastic collisions and/or weak SO interactions, the electronic process is the most efficient to convert the hydrogen by the emission of triplet excitons. On the contrary, when the collision lasts sufficiently to couple singlet and triplet electron states: τ≫T, the nuclear process is more efficient. Note that the conversion time for the (*e*) and (*n*) channels has opposite dependence on the scattering correlation time: $t_{op}^{\left(e\right)}∼\tau$ and $t_{op}^{\left(n\right)}∼\tau^{-1}$, and thus different functions of the temperature.

Sugimoto & Fukutani [23] first suggested the nuclear process for its efficiency to convert the hydrogen adsorbed on solid water. It was argued that the SO interaction could be effective inside the molecule because of a peculiar level crossing enhanced by a giant and inhomogeneous surface electric field. Our description in terms of electron repulsion associated to SO excitations elaborates that channel differently in taking into account local electron density.

The conversion algebra described in §4 is quite general and applies equally well to metals, semi-metals, semiconductors or insulating solids. The fast conversion rates, observed on Ag, were interpreted by an electron density outside the surface (increased by image charges), modelled by a smooth exponential decrease of 1 Å scale. The overlap with molecular electrons is not negligible, nor is their mutual repulsion. But relatively fast rates were also observed on the semi-metallic (pyrolysed) graphite [18]. It might be interesting to observe whether the dumping of the semiconductors by an important concentration of acceptor or donor impurities would speed the conversion rates.

Differently, the qualitative discussion of the electromagnetic channels of §5 is directed towards insulating catalysts, at low temperatures. Most of the insulators or semiconductors, where conversion was measured by optical means, have small gaps but fall into two different categories: those converting in a few minutes and the others in many hours. Very long patterns of many days have important applications for the conservation of pure para-hydrogen, for experimental research, medical imaging or liquid storage. For the category of hours, the Si and ZnO samples display an interesting variety of patterns with conversion times between 7 and 230 h [21]. Numerical simulations of hydrogen molecules in Si T_d_ interstitial sites indicate a slight charge transfer of 0.1 electron/H atom and a resulting elongation of the internuclear distance [38]. At the opposite range, ionic samples such as ASW and MOF containing many oxygen or zinc surface ions concentrate on their conversion times around a few minutes. They are characterized by surface dangling and unsaturated ligands, have a much larger electron extension outside the surface and approach the metallic character. It was a striking experimental fact to observe that the conversion of hydrogen molecules in front of O^2−^ ions was as fast as that felt by those in front of Zn^2+^ ions. Conversion does not distinguish between positive and negative charge distributions nor between attractive or repulsive main interaction. A recent and promising study of hydrogen in a porous polymer, by Raman spectrum and correlated to charge density by synchrotron radiation X-ray, has observed fast conversion at some non-equilibrium sites where the molecules experience huge and inhomogeneous electric fields [1].

The ‘excitonic’ charge extension outside the surface was compared qualitatively in figure 5 to the molecular one. Considering the spatial parameters, the closed shells covalent compounds convert the hydrogen in many hours, whereas the open ionic ones need a few minutes. Our model has the drawback of considering the thermal relaxation of the nuclear degrees of freedom in contact with an electron system at zero temperature, a full valence band and an empty conduction one. It is therefore impossible to deduce the conversion temperature dependence. Other approximations that limit that study include: the choice of a single correlation function for the hydrogen motion in the solid potential, and for the numerical estimates, the exponential form leads to a spectral density decreasing as the squared inverse of the excitation energy. Therefore, our criterion to decide between (*e*) and (*n*) channels remains qualitative.

The conversion reaction paths, herewith described, remind us of the triplet–triplet annihilations observed in the phosphorescent and delayed fluorescent phenomena (triplet formation and annihilation) occurring in radical pairs. The rapid variation of the surface inhomogeneous electrostatic field felt by the molecule replaces the electromagnetic radiation of the chemically induced nuclear and electron polarization (CIDNP and CIDEP) [45–47]. The nature and origin of these phenomena are, however, different, chemical (and light) for luminescence and isomer enrichment for hydrogen conversion.

When the phonon relaxation of the triplet excitons is delayed by a bottleneck, some emission of light might be induced by the hydrogen conversion and observed. Other experimental studies should also be of interest such as the optical pumping to the conduction band in order to transfer electrons towards the molecule through the antibonding bridge.

## Supplementary Material

Electronic Structures Nuclear Structures Electro-nuclear spin flows

## Data accessibility

All the research materials supporting the results in the article (notations, functions, operatorial elements and additional details of calculation) have been uploaded in the article's electronic supplementary material.
