# Supplementary material for: Nuclear conversion theory: molecular hydrogen in non-magnetic insulators
Source: R Soc Open Sci. 2016 Sep 7;3(9):160042. doi: 10.1098/rsos.160042 (PMC5043298; doi:10.1098/rsos.160042)
Supplement: Electronic Structures Nuclear Structures Electro-nuclear spin flows [file rsos160042supp1.docx]

**Annexes:**

**A1. Electronic Structures**

**A1.1 Spin-Orbital Eigenbasis and Energies**

Table A1: Electron Complex: Neutral and Ionic Antibonding Excited States

Table A2 : Molecule-Solid Excitation Energies

Table A3: Electron Repulsion: Non-Diagonal Coulomb Couplings

**A1.2 Electron Spin : Operators and Properties**

Table A4 : Spin-Orbit Singlet-Triplet Couplings

**A2. Nuclear Structures**

**A2.1 Molecular Rotation**

**A2.2 Nuclear Spin**

Table A5 : Hyperfine Contact Couplings

**A3. Electro-nuclear spin flows**

**A3.1 Repulsion-Contact: CY Channels**

**A3.2 Spin-Orbit-Repulsion-Contact: SOCY Channels**

Table A6: Spin-orbit paths in the solid

Table A7: Molecule-Solid Spin-orbit paths

**A3.3 The “excitonic” extension outside the solid**

Table A8 : Coulomb-Contact Integrals : Exponential Tails

**Glossary**

***Experimental Methods***

**EELS** Electron Energy Low Spectroscopy

**NMR** Nuclear Magnetic Resonance

**IR** Infrared Spectroscopy

**REMPI** Resonance Enhanced Multi Photon Ionization

**STM** Surface Scanning Tunneling Microscopy

**CIDN(E)P** Chemically Induced Dynamic Nuclear (Electron) Polarization

***Conversion Processes and Channels***

**CY: C**oulomb-h**Y**perfine contact : **XY, UY, VY**

**SOCY**: **S**pin-**O**rbit-**C**oulomb-h**Y**perfine contact : **SOXY, SOUY, SOVY**

(**C=X** exchange, **C=U** transfer to the molecule, **C=V** transfer to the solid)

**SOPY**: **S**pin-**O**rbit-di**P**olar -h**Y**perfine contact

(**P** is the electron dipolar momentum of the molecule that might couple to the solid Electric field)

**A1. Electronic Structures**

**A1.1 Spin-Orbital Eigenbasis and Energies**

The electron eigenbasis represented in the Fig. 2 is detailed in Table A1, corresponding eigen-energies are given in Table A2 and Coulomb repulsions in Table A3.

Hydrogen Electron Eigenstates**:** Fundamental singlet: ^1^Σ_g_ =│g$\bar{g}$│**.** The excited anti-bonding singlet: B^1^Σ_u_ = ^1^(gu) = (│g$\bar{u}$│ -│$\bar{g}u$│)/√2 and triplet$b{}^{3}{{}_{u}}$ eigenstates are described by molecular spin-orbitals g= (σ_g_1s) and u=( σ_u_*1s) calculated at a fixed inter-nuclear distance of R=1.4$a_{0}$ [48]. Upper bars denote a spin down. ^2^Σ_g_ denote the electron ground state of the ion H_2_^+^, left with one electron g and ^2^Σ_g_ H_2_^-^=│$g\bar{g}u$│. Note that for the odd ${}_{u}^{+}$ states the nuclear coupling is opposite to the even states: (J=0, I=1) and (J=1, I=0). In that way electron and nuclear symmetries are intricately linked, the ortho and para states combine differently space and spin, electron and nuclear manifolds.

Solid Eigenstates

The fundamental singlet ground state of the solid denoted: ^1^Г_0_ =│ξ$\bar{\xi}....$│is composed of all doubly occupied valence states ξ. The considered basis contains also neutral and ionic one-electron excited eigenstates: the triplet exciton where one electron is promoted to a state of the conduction band: │ξ$. ..$│, with parallel spins; a full VB to which one conduction electron is added: ^2^Г_-_ = $│..\xi\bar{\xi}.. │$and the valence band with one electron extracted: ^2^Г_+_ = $│..\xi.. │$

Molecule-Solid Eigenstates

The fundamental ground state of the Molecule-Solid electron complex is the antisymmetrized product of the separate eigenstates: ${}^{1}\mathbf{G}$=^1^(^1^Г_0_ x ^1^Σ_g_) =│ξ$\bar{\xi}.... g\bar{g}$│. Similarly one electron excited states are either obtained from solid excitations: ${}^{3}\boldsymbol{\epsilon}$= ^3^(${}^{3}\epsilon$ x ^1^Σ_g_) or molecular ones:${}^{\boldsymbol{3}}\boldsymbol{P}$ = ^3^(^1^Г_0_ x ^3^Σ_u_). The two electron excited state:^2S+1^(${}^{3}\epsilon$ x ^3^Σ_u_) consider a simultaneous solid-molecule excitation and is restricted to S=0 or 1. In particular the two-electron singlet: ${}^{\boldsymbol{1}}\boldsymbol{P}$ = ^1^(${}^{3}\epsilon$ x ^3^Σ_u_) allows important “mol-sol bridge” opportunities of tunneling. The positively ionized molecule H_2_^+^ (^2^Σ_g_ =$│$g >) couples to a full VB to which one conduction electron is added, building either a full singlet or triplet: ${}^{[S]}R$ = ^2S+1^(^2^Г_-_ x ^2^Σ_g_) of the [Solid^-^ x H_2_^+^] complex. Inversely the transfer of a valence electron to the molecule leads to the eigenstates ^[S]^(^2^Г_+_ x ^2^Σ_u_) of the of the [Solid^+^ x H_2_^-^] complex. The main electron determinantal eigenfunctions of our simplified basis are tabulated in Table A1. Additional ones included in the calculations play a complementary but minor role: ^1^Σ_u_ and ^3^(${}^{3}\epsilon$ x ^3^Σ_u_).

**Table A1: Electron Complex: Neutral and Ionic Antibonding Excited States**

| **Г** |  | **Singlets** | **Triplets** |
| --- | --- | --- | --- |
| **G** | ground | ${}^{1}\mathbf{G}$=^1^(^1^Г_0_ x ^1^Σ_g_) =│ξ$\bar{\xi}.... g\bar{g}$│ |  |
| $\boldsymbol{\epsilon}$ | exciton | ${}^{1}\boldsymbol{\epsilon}$=^1^(${}^{1}\epsilon$ x ^1^Σ_g_) ={│ξ$\bar{}. .. g\bar{g}$│-│$\bar{\xi}. .. g\bar{g}$│} /$\surd2$ | ${}^{3}\boldsymbol{\epsilon}$= ^3^(${}^{3}\epsilon$ x ^1^Σ_g_) _(m=1)_ =│ξ$. .. g\bar{g}$│ |
| **P** | Sol-Mol | ${}^{\boldsymbol{1}}\boldsymbol{P}$=^1^(${}^{3}\epsilon$ x ^3^Σ_u_) = $\{\vert\xi... \bar{g}\bar{u}\vert$ +$\vert\bar{\xi}\bar{}... gu\vert$}/√3 **-** {$\vert\bar{\xi}. .. g\bar{u}\vert$+$\left\vert\bar{\xi}... \bar{g}u \right\vert+\vert\xi\bar{}... g\bar{u}\vert$ +$\vert\xi\bar{}. .. \bar{g}u\vert\}/23$ | ${}^{\boldsymbol{3}}\boldsymbol{P}$ = ^3^(^1^Г_0_ x ^3^Σ_u_)_(m=1)_ = │ξ$\bar{\xi}..\mathrm{gu}$│ |
| **Q** | Sol^+^ Mol^-^ | ${}^{1}\mathbf{Q}$ = ^1^(^2^Г_+_ x ^2^Σ_u_)= {│ξ$\bar{u}... g\bar{g}$│-│$\bar{\xi}u\ldots g\bar{g}$│}/√2 | ${}^{3}\mathbf{Q}$ =^3^(^2^Г_+_ x ^2^Σ_u_)_(m=1)_ = │ξ$u... g\bar{g}$│ |
| **R** | Sol^-^Mol^+^ | ${}^{1}\boldsymbol{R}$ = ^1^(^2^Г_-_ x ^2^Σ_g_) ={│ξ$\bar{\xi}... \eta\bar{g}$│-│ξ$\bar{\xi}...\bar{}g$│}/$\surd2$ | ${}^{3}\boldsymbol{R}$ = ^3^(^2^Г_-_ x ^2^Σ_g_) _(m=1)_ = │ξ$\bar{\xi}... \eta g$│ |

The corresponding eigenenergies are given in Table A2. $\varepsilon_{g}$and $\varepsilon_{u}$are one electron energies whereas HF energies include Coulomb and exchange integrals: ${\varepsilon'}_{g}$ = $\varepsilon_{g}+J_{\mathrm{gg}}$ = - 15.8 eV; ${\varepsilon'}_{u}$ = $\varepsilon_{u}$ + $J_{gu}$ - $K_{gu}$ = 0.3 eV (with $\lambda$ = 1.189 a.u.). Note that (${}^{3}Q$) =$E({}^{1}Q)$ and E(${}^{3}R$) = $E({}^{1}R)$ but the singlet and triplet manifolds of the neutral P states have energies that differ by about the gap value ($\varepsilon_{\xi\eta})$.

**Table A2 : Molecule-Solid Excitation Energies**

| Excitation Energies | HF Energies |
| --- | --- |
| E(${}^{3}\epsilon$) – E(${}^{1}G$) = $\varepsilon_{\xi\eta}$ | ${\varepsilon'}_{\xi}$= $\varepsilon_{\xi}+ J_{\xi\xi}$+$\sum_{(\alpha\neq\xi)\in BV} \left( {2J}_{\alpha\xi}- K_{\alpha\xi} \right)$ |
| E(${}^{1}P$) – E(${}^{1}G$)=$\varepsilon_{\xi\eta}$ +$\varepsilon_{gu}$ | ${\varepsilon'}_{\eta}=\varepsilon_{\eta}+$+$\sum_{\alpha\in BV} \left( {2J}_{\alpha\eta}- K_{\alpha\eta} \right)$ |
| E(${}^{3}P$) – E(${}^{1}G$)$=\varepsilon_{gu}=$ ${\varepsilon'}_{u}- {\varepsilon'}_{g}$ | $\varepsilon_{\xi\eta}={\varepsilon'}_{\eta}-{\varepsilon'}_{\xi}$+ $J_{\xi\eta}- K_{\xi\eta}$ |
| E(${}^{3}Q$) – E(${}^{1}G$)= $\varepsilon_{\xi u}$= ${\varepsilon'}_{u}- {\varepsilon'}_{\xi}$ | ${\varepsilon'}_{g}$ = $\varepsilon_{g}+ J_{\mathrm{gg}}$ |
| E(${}^{3}R$) – E(${}^{1}G$)= $\varepsilon_{g\eta}$= ${\varepsilon'}_{\eta}- {\varepsilon'}_{g}$ | ${\varepsilon'}_{u}$ = $\varepsilon_{u}$ + $J_{gu}$ - $K_{gu}$ |

The molecule-solid electron repulsions are tabulated in Table A3, defining also the terminology of the C = X, U, V Coulomb repulsive interactions. The singlet channels connect the ground state to the excited singlets while the triplet ones connect the excitonic magnetic state to the excited charge transfer triplets. The rotational operator arising from the link between the molecular and solid frames is extracted whenever it appears in the molecular antibonding u state, as defined in A2.1.

**Table A3: Electron Repulsion: Non-Diagonal Coulomb Matrix Elements**

|  | Singlet Repulsion | Triplet Repulsion |
| --- | --- | --- |
| $\boldsymbol{\Gamma}$ | $\left\langle{}^{\boldsymbol{1}}\mathbf{G} \vert\mathbf{C} \vert{}^{\boldsymbol{1}}\boldsymbol{\Gamma} \right\rangle$ | $\left\langle{}^{\boldsymbol{3}}\boldsymbol{\epsilon} \vert\mathbf{C} \vert{}^{\boldsymbol{3}}\boldsymbol{\Gamma} \right\rangle$ |
| **P** | $\sqrt{3}<\xi│\frac{1}{r_{12}}│u^{1}g>$.$\boldsymbol{Y}^{1}\left( \hat{ab} \right)$  =${(\sqrt{6}) x^{1}}_{\eta\xi}.\boldsymbol{Y}^{1}\left( \hat{ab} \right)$ | -$<\xi│\frac{1}{r_{12}}│gu^{1}>.\boldsymbol{Y}^{1}(\hat{ab})$=  $-{(\sqrt{2}) x^{1}}_{\xi\eta}.\boldsymbol{Y}^{1}\left( \hat{ab} \right)$ |
| **Q** | ${2U^{1}}_{\xi u}.\boldsymbol{Y}^{1}\left( \hat{ab} \right)=\boldsymbol{Y}^{1}\left( \hat{ab} \right).$  $\sqrt{2}\left\{ 2<g\xi│\frac{1}{r_{12}}│gu^{1}>+<\xi\xi│\frac{1}{r_{12}}│\xi u^{1}> \right\}$ | $\sqrt{2} {U^{1}}_{\eta u}.\boldsymbol{Y}^{1}\left( \hat{ab} \right)= \boldsymbol{Y}^{1}(\hat{ab})$.$\begin{aligned} \\ \left\{ 2<g│\frac{1}{r_{12}}│gu^{1}>+<\xi│\frac{1}{r_{12}}│\xi u^{1}> \right\} \end{aligned}$ |
| **R** | ${2 V}_{g\eta}=\sqrt{2}\left\{ \begin{aligned} 2<\xi g│\frac{1}{r_{12}}│\xi> + \\ <gg│\frac{1}{r_{12}}│g> \end{aligned} \right\}$ | ${\sqrt{2}V}_{g\xi}$= $<\xi g│\frac{1}{r_{12}}│\xi\xi>+$  $<g│\frac{1}{r_{12}}│\xi> + <gg│\frac{1}{r_{12}}│g\xi>$ |

The exchange operator is denoted:${X^{1}}_{\xi\eta}$=$<\xi+\xi│\frac{1}{r_{12}}│u^{1}g>$ = $\frac{\left( {x^{1}}_{\eta\xi}+{x^{1}}_{\xi\eta} \right)}{\sqrt{2}}$ .

**A1.2 Electron Spin : Operators and Properties**

The spin singlet-triplet tensor is defined by the difference between the spin momenta of two electron spins (1/2) :$\boldsymbol{\sigma}^{\boldsymbol{1}}\boldsymbol{=}\boldsymbol{s}\left( \boldsymbol{1} \right)\boldsymbol{-}\boldsymbol{s}\boldsymbol{(}\boldsymbol{2}\boldsymbol{)}$**;** or by its reduced matrix element : $\left\langle0 | \left| \sigma^{1} \right| | s=1 \right\rangle$=$\sqrt{3}$.

It has the properties: $\sum_{m} \left\langle0 | \sigma_{\alpha}^{1} | s=1m \right\rangle\left\langle s=1m | \sigma_{\beta}^{1} | 0 \right\rangle$ =$3 \delta_{\alpha,-\beta}.$and $\left\langle0 | \boldsymbol{\sigma}^{\boldsymbol{1}}\boldsymbol{.}\boldsymbol{\sigma}^{\boldsymbol{1}} | 0 \right\rangle$= 3.

**Spin Orbit Couplings: SOC**

The transformation of the relativistic many electron Breit equation produces two terms, which contribute to spin-orbit effects:

**V**_SO_ **=**  $\frac{\alpha^{2}}{2} \sum_{\alpha} \left\{ \mathbf{L}(\alpha)+\sum_{\beta<\alpha} \mathbf{L}(\alpha,\beta) \right\}. \mathbf{s}(\alpha)$

The one-body Hamiltonian is expressed in terms of orbital operators, functions of the nucleus n and electron $\alpha$: $\mathbf{L}(\alpha)= -\sum_{n} \boldsymbol{\nabla}\left( \frac{Z_{n}}{r_{\alpha n}} \right)\times\mathbf{p(}\alpha)$, whereas the two-body operators link electron pairs: $\mathbf{L}(\alpha,\beta)= \boldsymbol{\nabla}\left( \frac{1}{r_{\alpha\beta}} \right)\times\left[ \mathbf{p}\left( \alpha\right)- \mathbf{p}(\beta) \right]$. That magnetic coupling is active inside the molecules and the solid but can also link molecular and solid eigenstates. Within the approximate mean field model, each electron feels an average polarization field: $\boldsymbol{\Lambda}$ = $\sum_{\alpha} \boldsymbol{\Lambda}(\alpha).\mathbf{s}(\alpha)$. The couplings G-$\Gamma$ involve interactions between bonding (b) and antibonding (ab) orbitals: (b-ab) either “mol-mol” (gu) or “mol-sol” ($\xi$u and g$\eta$) or “sol-sol” ($\xi\eta$). The couplings $\Gamma$ -$\Gamma$ involve interactions that are all internal to the solid, either “b-ab”($\xi\eta$) or ”b-b” ($\xi\xi$) or “ab-ab” ($\eta\eta$). Most of the matrix elements are tabulated in Table A4.

**Table A4 : Spin-Orbit Singlet-Triplet Matrix Elements**

| $\Gamma$ | Solid | Molecular and Molecule-Solid |
| --- | --- | --- |
|  | $\boldsymbol{\Lambda}\left( \Gamma\Gamma\right)$= $\left\langle{}^{3}\Gamma\vert\Lambda\vert{}^{1}\Gamma) \right\rangle$=$\boldsymbol{\Lambda}\left( \gamma\gamma' \right)$.$\boldsymbol{\sigma}$ | $\boldsymbol{\Lambda}\left( G\Gamma\right)$=$\left\langle{}^{1}G \vert\Lambda\vert{}^{3}\Gamma\right\rangle$=$\boldsymbol{\Lambda}\left( \gamma\right)$.$\boldsymbol{\sigma}$ |
| P | (1/$2\sqrt{6}$)$\boldsymbol{\sigma.}\boldsymbol{\Lambda}(\xi\eta)$ | $\mathbf{-(}1/\sqrt{2}\boldsymbol{)\sigma.}\boldsymbol{\Lambda}(gu)$ |
| Q | $\mathbf{(}1/2\boldsymbol{)\sigma.}\boldsymbol{\Lambda}(\xi\xi)$ | $\mathbf{-(}1/\sqrt{2}\mathbf{)}$ $\boldsymbol{\sigma.}\boldsymbol{\Lambda}(\xi u)$ |
| R | $\mathbf{(}1/2\boldsymbol{)\sigma}\mathbf{.}\boldsymbol{\Lambda}(\eta\eta)$ | $\mathbf{-(}1/\sqrt{2}\mathbf{)}$ $\boldsymbol{\sigma}\mathbf{.}\boldsymbol{\Lambda}(g\eta)$ |
| E | $\mathbf{(}1/2\mathbf{)}\left( \boldsymbol{\sigma}\mathbf{.}\boldsymbol{\Lambda}\left( \xi\xi\right)-\boldsymbol{\sigma}\mathbf{.}\boldsymbol{\Lambda}\left( \eta\eta\right) \right)$ | **-** $\mathbf{(}1/\sqrt{2}\boldsymbol{)\sigma}\mathbf{.}\boldsymbol{\Lambda}(\xi\eta)$ |

$\boldsymbol{\Lambda}\left( Q\boldsymbol{\epsilon} \right)$=$\left\langle{}^{3}Q | \Lambda| {}^{1}\boldsymbol{\epsilon}) \right\rangle$= -$\mathbf{(}1/2\mathbf{)}\boldsymbol{\sigma}\mathbf{.}\boldsymbol{\Lambda}(u\eta)$ and $\boldsymbol{\Lambda}\left( R\boldsymbol{\epsilon} \right)$=$\left\langle{}^{3}R | \Lambda| {}^{1}\boldsymbol{\epsilon}) \right\rangle$= -$\mathbf{(}1/2\mathbf{)}\boldsymbol{\sigma}\mathbf{.}\boldsymbol{\Lambda}(\xi g)$

**A2. Nuclear Structures**

**A2.1 Molecular Rotation**

**Energies and Populations**

In the electronic ground state of the hydrogen molecule, the ortho and para states combine the nuclear symmetries such as the rotational and spin states: ortho(J odd, I=1) and para(J even, I=0). The para and ortho states are defined by the following quantum numbers: ${j_{p}m_{p}, j}_{o}m_{o}i_{o}$. The total partition function is defined by $:z= \sum_{\alpha} z_{\alpha}=$ $\sum_{\alpha} ge^{-\frac{\varepsilon_{\alpha}}{kT}}.$ It is however interesting to divide the rotational space in two subspaces corresponding to the two varieties v = {o, p} of related partition functions z_v_ = {z_o_, z_p_} such as $z_{v}= \sum_{\alpha\in v} z_{\alpha}$ and z = z_o_ + z_p_. The rotational energies and nuclear degeneracies are: E_J_= BJ(J+1) and g_α_ =(2I+1)(2J+1), when the rotation is free. The total ortho concentration o =$\sum_{\alpha\in o} n_{\alpha}$ and similarly for the para one when the summation is performed on para states: $\alpha\in p$. The irreversible flow towards equilibrium, $n_{\alpha}(T)=\frac{z_{\alpha}}{z}$, is conveniently described by the master equation for populations which governs the time evolution of the hydrogen populations $n_{\alpha}$, whose rotational state α is of energy $\varepsilon_{\alpha}$: $\frac{{dn}_{\alpha}}{dt}$ = $\sum_{\beta} W_{\alpha\beta}\left\{ \frac{n_{\beta}}{z_{\beta}}- \frac{n_{\alpha}}{z_{\alpha}} \right\}$.

**Rotation in antibonding manifold**

The odd antibonding molecular orbital u=1s(a)- 1s(b) might be referred to the “lab” frame: $u\left( \boldsymbol{r} \right)$ = u(r)$\mathbf{Y}^{1}\left( \hat{r} \right)$.$\mathbf{Y}^{1}(\hat{ab})$. By defining 3 orbitals of 2p character u_x_, u_y_, u_z_ in cartesian coordinates or in spherical basis : $u^{1}\left( \boldsymbol{r} \right)$ = u(r)$\mathbf{Y}^{1}\left( \hat{r} \right)$. It is possible to express u as the scalar product between the electron function and the spherical harmonic of the internuclear orientation : $u\left( \boldsymbol{r} \right)$ = $u^{1}\left( \boldsymbol{r} \right)$.$\mathbf{Y}^{1}(\hat{ab})$, whose o-p rotational matrix elements are :

$\left\langle j_{p}m_{p} | u\left( \boldsymbol{r} \right) | j_{o}m_{o} \right\rangle$= $a_{op} u_{m_{op}}^{1}\left( \boldsymbol{r} \right)$

where $m_{op}=m_{o}-m_{p}= 0,\pm1$_,_ and the rotational constant $a_{op}$ being defined as:

$a_{op}=\left\langle j_{p}m_{p} | Y_{m_{p}-m_{o}}^{1} | j_{o}m_{o} \right\rangle=\binom{j_{p} 1 j_{o}}{m_{p} m_{o}-m_{p} m_{o}}\binom{j 1 j}{0 0 0}$_;_ in particular : a_01_ = $\sqrt{2}$.

**A2.2 Nuclear Spin**

**Operators and Properties**

The nuclear spin singlet-triplet tensor is defined as the electron one, either by the difference between the spin momenta of two nuclear spins (1/2) : $\boldsymbol{i}^{\boldsymbol{1}}\boldsymbol{=}\boldsymbol{i}\left( a \right)\boldsymbol{-}\boldsymbol{i}\boldsymbol{(}b\boldsymbol{)}$**;** or by its reduced matrix element $\left\langle0 | \left| i^{1} \right| | I=1 \right\rangle$=$\sqrt{3}$ and $\left\langle0 | i_{\alpha}^{1} | 1i_{o} \right\rangle$ **=** $\delta_{\alpha,-i_{o}}$. Table A5 gives the Hyperfine Contact Matrix Elements in terms of electron densities in molecular space. The spin operators $\mathbf{i}$ and $\boldsymbol{\sigma}$ are extracted.. Additional hyperfine couplings included in the following are: $\left\langle{}^{1}G | Y | {}^{3}\Gamma\right\rangle$ $= \mathbf{Y}\left( G\Gamma\right)= \mathbf{Y}\left( \gamma\right) \mathbf{i}\mathbf{.}\boldsymbol{\sigma}$ and $\left\langle{}^{3}\epsilon| Y | {}^{1}\Gamma\right\rangle$ $= \mathbf{Y}\left( \epsilon\Gamma\right)= \mathbf{Y}\left( \epsilon\gamma\right) \mathbf{i}\mathbf{.}\boldsymbol{\sigma}$**.**

| $\mathbf{Y}\left( \mathrm{gu} \right)$= ζ u(a) g(a) | $\mathbf{Y}\left( \xi u \right)$= ζ u(a)  $\xi$ (m) | $\mathbf{Y}\left( u \right)$= $u(a)$  $(m)$ | $\mathbf{Y}^{1}\left( g\xi\right)$= ζ g(a)  $\mathrm{ab}_{v}\boldsymbol{\nabla}^{\mathbf{1}}\xi$ (m) | $\mathbf{Y}^{1}\left( g \right)$= $g\left( a \right)$  $\mathrm{ab}_{v}\boldsymbol{\nabla}^{\mathbf{1}}(m)$ |
| --- | --- | --- | --- | --- |

where $\mathrm{ab}_{v}=ab\sqrt{\frac{4\pi}{3}}$ . m denotes the molecule center and **a**, **b** the 2 protons.

**Table A5 : Hyperfine Contact Matrix Elements**

| $\Gamma$ | $\sqrt{2}\mathbf{Y}\left( \gamma\right)$ | 2 $\mathbf{Y}\left( \epsilon\gamma\right)$ |
| --- | --- | --- |
| P | ${- Y}_{\mathrm{gu}}$ | $Y_{\mathrm{gu}}\sqrt{\frac{2}{3}}$ |
| Q | $- Y_{\xi u}$ | ${- Y}_{u}$ |
| R | $\boldsymbol{Y}^{1}\left( \hat{ab} \right) {\mathbf{.Y}^{\mathbf{1}}}_{g}$ | $\boldsymbol{Y}^{\boldsymbol{1}}\left( \hat{ab} \right) {{.\mathbf{Y}}^{1}}_{g\xi}$ |

**A3. Electronuclear spin flows**

Channels and Tunneling Paths : $℘ \equiv\left\{ \left( e, n \right), \left( \xi,\eta\right), \left( X, U, V \right) \right\}\left\{ j_{p},m_{p}; j_{o},m_{o} \right\}$.

The Hyperfine Transition Strength gives micro-reversible o-p transition probabilities between the two manifolds: ${j_{p}m_{p}, j}_{o}m_{o}i_{o}$: W ($j_{o},j_{p}$)= $\sum_{m_{op},i_{0}} W \left( j_{o},j_{p},m_{op}{,i}_{0} \right)$**.** The summation is performed over the nuclear spin and rotational magnetic quantum numbers $m_{o},m_{p}\ldots$ as well as over all accessible final electronic states. The thermostat absorbs the o-p energy.

**A3.1 Repulsion-Contact: CY Channels**

Any double singlet-triplet transition between the spin manifolds of the protons (o$\leftrightarrow$p) and those of the electrons (S$\leftrightarrow$T) « crosses » virtual intermediate states of either singlet or triplet spin:

$\left\langle I=1,S=0 | \mathbf{C} \mathbf{G}_{\boldsymbol{0}}\mathbf{Y}\mathbf{+}\mathbf{Y} \mathbf{G}_{\boldsymbol{1}}\mathbf{C} | I=0,S=1 \right\rangle$ = $\sum_{\Gamma} \frac{\left\langle I=1,S=0 | \mathbf{Y} | \Gamma, I=0,S=1 \right\rangle\left\langle\Gamma, I=0,S=1 | \mathbf{C} | I=0,S=1 \right\rangle}{\varepsilon_{\Gamma}}$ +

$$\sum_{\Gamma} \frac{\left\langle I=1,S=0 | \mathbf{C} | \Gamma,I=1 S=0 \right\rangle\left\langle\Gamma,I=1, S=0 | \mathbf{Y} | I=0,S=1 \right\rangle}{\varepsilon_{\Gamma}}$$

**Properties :** O is hermitian: $\left\langle G;p | O | G;o \right\rangle$ = $\left\langle G;o | O | G;p \right\rangle^{*}$

$$\left\langle G ;p | \mathbf{V} | \xi\eta, s=1;o \right\rangle=\left\langle p; \xi\eta, s=1 | \mathbf{V} | G;o \right\rangle^{*}$$

$$\left\langle j_{p}m_{p};I=S=0 | \mathbf{C}\mathbf{G}_{\boldsymbol{0}}\mathbf{Y + Y}\mathbf{G}_{\boldsymbol{1}}\mathbf{C} | {\xi; j}_{o}m_{o};I=S=1,m_{i} m_{s} \right\rangle=\sqrt{2}\left\{ a_{op} \left\langle I=S=0 | \mathbf{i}\mathbf{.} | I=S=1,m_{i} m_{s} \right\rangle\right\}\left\{ \frac{\mathrm{CY}}{\Phi} \right\}_{m_{op},\xi\eta}$$

**A3.2 Spin-Orbit-Repulsion-Contact: SOCY Channels**

Different forms of the orbital SOCY tensor $\left\{ \boldsymbol{\lambda}\mathbf{.C. y} \right\}$ can be used to express the transfer of the nuclear momenta to the electron system. In section 3.c the operator was written in spherical components $\sum_{\xi\eta} \left[ \left\{ \boldsymbol{\lambda} \right\}_{i_{o}}^{1}{. \left\{ \mathbf{C}y \right\}}_{m_{op}}^{1} \right]\left( \xi\eta\right)$ and the squared sum along 3 perpendicular directions. The components products might also be recoupled to form tensors of different ranks k=0, 2. That has the advantage to separate and figure scalar products of electron orbital averages and nuclear spin rotation operators:

$$\left\langle G | \boldsymbol{O} | G \right\rangle= \sum_{℘, k=0,2} {c_{k}\left[ \left\{ \boldsymbol{\lambda} \right\}^{1}\times\left\{ \mathbf{C} y \right\}^{1} \right]}^{k}\left( ℘ \right) .\left[ \mathbf{i}^{\boldsymbol{1}}\boldsymbol{\times}Y^{1}\left( \hat{ab} \right) \right]^{\boldsymbol{k}}$$

In the following we omit the tensorial forms and review the orbital content of these tensors as related to the different electron paths able to convert the hydrogen. The main “excitonic” **SOCY** paths were described in the text by all possible repulsion-contact:$\boldsymbol{\epsilon}(\xi\to\eta)$ transitions from the valence band to the conduction band of energy $\varepsilon_{\xi\eta}$, provided that the spin-orbit interaction binds together the excited electron and hole left behind to form a triplet spin. It sums singlet and triplet channels:

$\left\{ \boldsymbol{\lambda}\mathbf{.C. y} \right\}\left( \xi\eta\right)$ = $\left\langle{}^{1}G | \boldsymbol{\Lambda} | {}^{3}\epsilon\right\rangle\sum_{\Gamma} \left\{ \left\langle{}^{3}\epsilon| \mathbf{C} | {}^{3}\Gamma\right\rangle\left\langle{}^{3}\Gamma| \mathbf{Y} | {}^{1}G) \right\rangle+\left\langle{}^{3}\epsilon| \mathbf{Y} | {}^{1}\Gamma\right\rangle\left\langle{}^{1}\Gamma| \mathbf{C} | {}^{1}G \right\rangle\right\}/{{\varepsilon_{\xi\eta}\varepsilon}_{\Gamma}}$

Other channels are possible. The following one: $\mathbf{Y}.G_{1}.\boldsymbol{\Lambda}.G_{0}.\mathbf{C}$ considers that the repulsion and the hyperfine contact connects separately the ground state to different spin manifolds of the same orbital content $\Gamma$. The SO links then all excited singlet-triplet $({}^{1}{\Gamma-{}^{3}\Gamma})$knots. The corresponding tensor:

$$\sum_{\Gamma} {\left\langle{}^{1}G | \mathbf{Y} | {}^{3}\Gamma\right\rangle\left\langle{}^{3}\Gamma| \boldsymbol{\Lambda} | {}^{1}\Gamma\right\rangle\left\langle{}^{1}\Gamma| \mathbf{C} | {}^{1}G) \right\rangle}/{E({}^{3}\Gamma)E({}^{1}\Gamma)}$$

combines the matrix elements of Table A6:

**Table A6: Spin-orbit paths in the solid**

|  | $\left\langle{}^{1}G \vert\mathbf{Y} \vert{}^{3}\Gamma\right\rangle$ | $\left\langle{}^{3}\Gamma\vert\boldsymbol{\Lambda} \vert{}^{1}\Gamma\right\rangle$ | $\left\langle{}^{1}\Gamma\vert\mathbf{C} \vert{}^{1}G) \right\rangle$ | $E({}^{3}\Gamma)E({}^{1}\Gamma)$ |
| --- | --- | --- | --- | --- |
| P | Y(gu) | $\boldsymbol{\Lambda}(\xi\eta)$ | ${x^{1}}_{\eta\xi}.\boldsymbol{Y}^{1}\left( \hat{ab} \right)$ | ${(\varepsilon}_{\xi\eta}$ +$\varepsilon_{gu})\varepsilon_{gu}$ |
| R | $\boldsymbol{Y}^{1}\left( \hat{ab} \right). \mathbf{Y}^{1}(g)$ | $\boldsymbol{\Lambda}(\eta\eta)$ | V($g\eta)$ | ${\varepsilon_{g\eta}}^{2}$ |

The first term corresponding to $\Gamma=P$ and containing the non-diagonal spin-orbit valence-conduction coupling $\Lambda\left( \xi\eta\right)$ complements the main ones but remains a weak correction because it is divided by the square of the highest energy $\varepsilon_{\mathrm{gu}}$. The second term has been omitted since it contains the almost vanishing valence band SO coupling: $\left\{ \boldsymbol{\lambda}\mathbf{.C. y} \right\}\left( \mathrm{VB} \right)$= $\frac{U^{1}\left( \xi u \right)Y_{\xi u}\left\langle\xi| \Lambda^{1} | \xi\right\rangle}{{\varepsilon_{\xi u}}^{2}}$. The third term is more important, it depends on the conduction band SO and contact couplings and a virtual molecule solid transfer:

$\left\{ \boldsymbol{\lambda}\mathbf{.C. y} \right\}\left( \mathrm{CB} \right)= \frac{\left\langle\eta| \boldsymbol{\Lambda}^{1} | \eta\right\rangle Y_{g\eta} V_{g\eta}}{{\varepsilon_{g\eta}}^{2}}$.

Finally we consider the spin-orbit couplings inside the molecule and as a molecule-solid link.

$$\sum_{\Gamma} {\left\langle{}^{1}G | \boldsymbol{\Lambda} | {}^{3}\Gamma\right\rangle\left\langle{}^{3}\Gamma| \mathbf{C} | {}^{3}\epsilon\right\rangle\left\langle{}^{3}\epsilon| \mathbf{Y} | {}^{1}G) \right\rangle}/{E({}^{3}\Gamma)E({}^{3}\epsilon)}$$

which combines the matrix elements of Table A7:

**Table A7: Molecule-solid spin-orbit paths**

|  | $\left\langle{}^{1}G \vert\boldsymbol{\Lambda} \vert{}^{3}\Gamma\right\rangle$ | $\left\langle{}^{3}\Gamma\vert\mathbf{C} \vert{}^{3}\epsilon\right\rangle$ | $\left\langle{}^{3}\epsilon\vert\mathbf{Y} \vert{}^{1}G) \right\rangle$ | $E({}^{3}\Gamma)\varepsilon_{\xi\eta}$ |
| --- | --- | --- | --- | --- |
| P | $\boldsymbol{\Lambda}(gu)$ | ${x^{1}}_{\xi\eta}.\boldsymbol{Y}^{1}\left( \hat{ab} \right)$ | **Y**($\xi\eta$) | ${(\varepsilon}_{\xi\eta}$ +$\varepsilon_{gu})\varepsilon_{\xi\eta}$ |
| Q | $\boldsymbol{\Lambda}(\xi u)$ | ${U^{1}}_{\eta u}.\boldsymbol{Y}^{1}\left( \hat{ab} \right)$ | **Y**($\xi\eta$) | $\varepsilon_{\xi u}\varepsilon_{\xi\eta}$ |
| R | $\boldsymbol{\Lambda}(g\eta)$ | V($g\xi)$ | **Y**($\xi\eta$) | $\varepsilon_{g\eta}\varepsilon_{\xi\eta}$ |

These paths can be considered to lead to conversion rates an order of magnitude slower than the main valence-conduction $℘\left( \xi\eta\right)$ and conduction-conduction $℘\left( \eta\eta\right)$ electron paths. All of these require weak simultaneous contacts of the valence and conduction electrons with the hydrogen protons.

**A3.3 The “excitonic” extension outside the solid**

The products of non-diagonal Coulomb integrals with contact amplitudes are classified in Table A8, in function of the number of molecule (m) and solid wave functions (s) that are coupled by the electron repulsion, the regions of preponderant integration spaces and their exponential tails.

**Table A8 : Coulomb-Contact Integrals : Exponentials Tails**

| COULOMB-CONTACT Integrals | Nature of Eigenstates | Molecular Space  (m) | Solid Space  (s) |
| --- | --- | --- | --- |
| $<\xi\eta│\frac{1}{r_{12}}│\mathrm{ug}>$ gu(a) | 2m + 2s | $e^{- \left( \xi+ \eta\right)r}$ | $e^{- (\lambda_{g}+ \lambda_{u})r}$ |
| $<\xi\xi││\xi u> \eta$(m)u(a)  $<\xi││\xi u>\xi$(m)u(a)  $<\xi\eta││\xi g> \xi(m)g(a)$  $<\xi\xi││\xi g> \eta$(m) g(a)  $<\xi││g>\eta$(m)g(a) | 1m + 3s | (negligible) | $e^{- \left( \eta+ \lambda_{u} \right)r}$  $e^{- \left( \xi+ \lambda_{u} \right)r}$  $e^{- \left( \xi+ \lambda_{g} \right)r}$  $e^{- \left( \lambda_{g}+ \eta\right)r}$  $e^{- \left( \lambda_{g}+ \eta\right)r}$ |
| $<gu││g\xi>\eta$(m) u(a)  $<gu││g\eta>\xi\left( m \right)u(a)$  $<gg││g\eta> \xi$(m) g(a)  $<gg││g\xi> \eta$(m) g(a) | 3m + 1s | $e^{- \left( \xi+ \eta\right)r}$ | (negligible) |

**Solid** $\boldsymbol{\leftrightarrow}$ **Molecule Exchanges**

The exchange integrals$X_{\xi}$ involve the repulsion between 2 mol and 2 solid mixed densities bonding and antibonding (b-ab) $\xi u and g$. That exchange integral can be divided in two parts one in the solid space decreasing as $e^{- (\lambda_{g}+ \lambda_{u})r}$ while the integration in molecular space decreases as ${\sim e}^{- \xi r- \eta r}$. Such an exchange is quite small, of the order of X= 10 -100 meV but it is enhanced by the largest hyperfine contact $Y_{\mathrm{gu}}$ between the protons and the molecular electrons: about 10^-6^ eV. At the contrary, it is decreased by a large excitation energy $\varepsilon_{\mathrm{gu}}$, around 10 eV. As already discussed in § 4c, $\varepsilon_{\mathrm{gu}}$might be reduced when the antibonding molecular u state is alloyed to the solid conduction states. In total the **XY**channel has an amplitude of about 10^-7^- 10^-9^ eV.

**Solid**$\boldsymbol{\to}$**Molecule Transfers**

For the **UY** channel through ionic excited states, both $U_{\xi} Y_{\eta u}{\cong U}Y_{\xi u}$ involve the mutual overlap of molecule-solid and ab-eigenstates. In molecular space the repulsion involves 3 molecule states and 1 solid state: $<g│\frac{1}{r_{12}}│gu>$, which when coupled to the contact of the solid tail with the protons, decreases as ${\sim e}^{- \xi r- \eta r}$. In solid space it involves 1 molecule and 3 solid states: $<\xi│\frac{1}{r_{12}}│\xi u>$, and leads to a decrease: ${\sim e}^{- \lambda r- \eta r}$.

$\varepsilon_{\xi u}$ is an excitation energy smaller than $\varepsilon_{\mathrm{gu}},$ but the contact ratio $Y_{\eta u}/Y_{\mathrm{gu}}$ is weak (5 10^-2^). In total the **UY**channel has an amplitude of about 10^-8^- 10^-10^ eV

**Molecule** $\boldsymbol{\to}$**Solid Transfers**

In the **VY**  channel the repulsion integrals are slightly smaller since only one ab-eigenstate contribute either in solid space $<\xi g││\xi\xi>$or in the molecular one : <gg││gη>. The contact terms $Y_{g\eta}$and $Y_{\eta u}$are similar in molecular space since the conduction states and their gradients have similar tails. However that channel is favored first because of a smaller energy excitation $\varepsilon_{g\eta}$ slightly larger than the gap value and might be enhanced either by an extension of the conduction states or by a closer approach of the molecule towards the solid. It is also favored by larger orbital contributions of the higher conduction states: $Y_{g\eta} V_{g\eta}$ ${\sim e}^{-2 \eta r}$. In total the **VY**channel has an amplitude of about 10^-6^- 10^-9^ eV.
